# Coarse-Graining Self-Assembly by the Stochastic Landscape Method

**DOI:** 10.1021/acs.jctc.5c01241

**Published:** 2025-10-27

**Authors:** Michael Faran, Gili Bisker

**Affiliations:** † School of Biomedical Engineering, Faculty of Engineering, 26745 Tel Aviv University, Tel Aviv 69978, Israel; ‡ The Center for Physics and Chemistry of Living Systems, Tel Aviv University, Tel Aviv 6997801, Israel; § The Center for Nanoscience and Nanotechnology, Tel Aviv University, Tel Aviv 6997801, Israel; ∥ The Center for Light-Matter Interaction, Tel Aviv University, Tel Aviv 6997801, Israel; ⊥ The Center for Computational Molecular and Materials Science, Tel Aviv University, Tel Aviv 6997801, Israel

## Abstract

Inferring the dynamics of many-body stochastic systems from data remains a fundamental challenge in statistical physics, particularly when models must provide both predictive power and physical interpretability. Nonequilibrium self-assembly, where molecular components are driven to form ordered structures from disordered building blocks, is a prime example, central to nanotechnology, material science, and biology. Markov state models (MSMs) have emerged as a powerful framework for representing and understanding the dynamic behavior of such complex systems by discretizing their high-dimensional phase space into a network of metastable states and transition probabilities. Yet, constructing accurate and interpretable MSMs for nonequilibrium self-assembly remains challenging, often requiring extensive, multidimensional data sets or system-specific assumptions. Here, we introduce a novel framework for constructing MSMs of nonequilibrium self-assembly based on the stochastic landscape method (SLM), a physically grounded approach previously shown to enable predictive control of assembly dynamics. Using a tractable amount of simulation data, our method effectively coarse-grains the vast state space into a low-dimensional model that accurately reproduces key dynamic observables, including yield and first assembly times, under both equilibrium and driven conditions. Furthermore, we show that the resulting MSM generalizes beyond the conditions used for their construction, enabling accurate predictions in previously unexplored physical parameter regimes, while reducing the computational cost of baseline simulation by several orders of magnitude. While developed in the context of nonequilibrium self-assembly, this approach is broadly applicable to many-body systems governed by stochastic dynamics, offering a general strategy for constructing interpretable, efficient models of complex processes.

## Graphical abstract

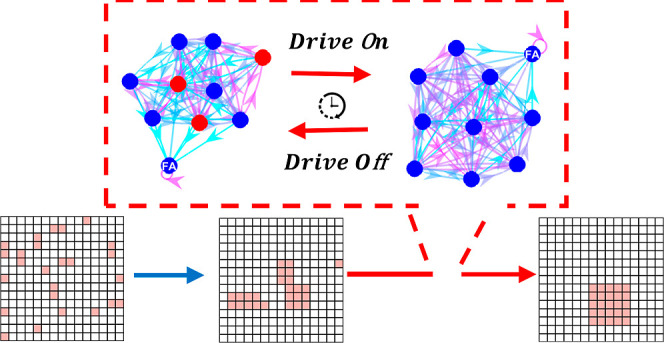

## Introduction

Self-assembly, the process by which molecular components organize into ordered structures from initially disordered building blocks, underpins a wide range of systems in biology,^1,2^ biomedical engineering,^3–5^ and materials science.^6–9^ The ubiquity of self-assembly across both natural and synthetic platforms has motivated extensive research efforts aimed at understanding its principles and developing strategies to actively control it. Nevertheless, directing or optimizing self-assembly remains a fundamental challenge in physics and engineering, owing to its complex, high-dimensional energy landscapes,^10,11^ its multifaceted design requirements,^12–15^ and the intrinsic trade-off between kinetic accessibility and thermodynamic stability.^16–18^

Recent studies have shown that introducing nonequilibrium driving through external forces, energy consumption, or environmental control can enhance assembly outcomes by improving yields, accelerating assembly kinetics, or enabling access to otherwise inaccessible structures. These effects have been demonstrated experimentally,^7,19–21^ validated through simulations,^17,22–25^ and supported by theoretical models.^26–29^ To harness the potential of external driving, various nonequilibrium control protocols have been developed. These include open-loop protocols, where the system is driven along predefined trajectories,^30–33^ and closed-loop strategies, where real-time feedback is used to modulate the driving based on the system’s evolving state.^11,34–38^

Beyond targeting specific structures or optimizing yields and assembly times, a central objective of self-assembly research is to uncover general physical principles that inform control strategies,^39,40^ enable biomedical and nanotechnology innovations,^3,5,41^ and reduce computational cost.^42^ Although self-assembling systems are inherently complex, recent advances in simulation and control have shown that key physically interpretable features of the process can be successfully extracted. Notable examples include the emergence of phase diagrams from nonequilibrium dynamics,^43,44^ time-complexity analyses of assembly pathways,^45^ observations of scale-invariant behavior,^46^ and quantification of memory time constants.^47^

Ultimately, a key goal in achieving physical interpretability is to derive coarse-grained descriptions of self-assembly in the form of emergent, time-dependent equations that capture the essential dynamics^48^ and reveal the underlying physical mechanisms. This objective reflects a growing trend in the physical sciences to extract reduced, interpretable models from complex simulation data using data-driven methodologies.^49^ In this context, surrogate models serve as efficient stand-ins for high-dimensional stochastic simulations while preserving interpretability.^50–53^ Such models can significantly reduce computational cost while providing deeper insight into the governing principles underlying the system dynamics. In the context of self-assembly, coarse-grained kinetic theories that produce effective, low-dimensional descriptions of system dynamics have been developed based on simulation data for specific systems.^29,46,54,55^ A prominent example is the construction of effective Smoluchowski equations for colloidal assembly by projecting the system dynamics onto estimated two-dimensional reaction coordinates.^56^ Such reduced descriptions have also been used to design external controls that steer the assembly outcomes.^57^

One of the simplest yet most elegant frameworks for describing emergent dynamics is the Markov state model (MSM).^58–62^ In this approach, the system’s vast phase space is discretized into a network, where each node in a graph represents a coarse-grained state of the system, while the directed edges correspond to transition probabilities defined by a transition matrix. MSMs offer a powerful tool for uncovering metastable states and understanding long-time scale dynamics from high-dimensional data. Nevertheless, constructing reliable MSMs presents several challenges. In particular, it is often unclear to what extent a given coarse-graining scheme exhibits dynamics that are sufficiently Markovian (memoryless), and limitations in sampling can further compromise model accuracy. These challenges, however, can often be mitigated through careful selection of time–lag parameters and rigorous statistical preprocessing techniques.^42^

MSMs have been proposed as predictive surrogates for self-assembly dynamics in both equilibrium settings^47,63–67^ and nonequilibrium contexts.^40,42,68,69^ These models have successfully predicted assembly yields, identified kinetic bottlenecks, and revealed distinct mechanistic pathways, offering physically interpretable insights into complex self-assembly behavior. A key advantage of MSMs lies in their ability to extrapolate system dynamics beyond directly sampled time scales, thereby reducing computational cost.^42^

MSMs are typically constructed by defining discrete system states based on structural descriptors, such as bond or cluster counts,^42^ bonding graphs,^47,64^ or spatial coordinates.^69^ For example, viral capsid models define states by clustering over structural similarity,^47^ whereas colloidal systems often rely on order parameters.^67^ These methods have enabled detailed insights into large assemblies composed of uniform subunits. In smaller or more heterogeneous systems, where stochasticity plays a central role,^70,71^ there remains an opportunity to explore alternative state decompositions based on low-dimensional, system-agnostic observables.

Recent advances have extended MSMs to model nonequilibrium systems by introducing additional layers of control and analysis. Grover et al.^72^ incorporated a Markov decision process framework into MSMs, requiring the definition of a reward function and the solution of a dynamic programming problem to identify optimal control policies. Trubiano and Hagan^40^ construct a series of MSMs at discrete control parameters and interpolate between them to simulate time-dependent driving, which requires both parameter-resolved simulations and matrix interpolation schemes. These developments open new doors for protocol design, involving additional layers of modeling, such as reward-based optimization or parameter-resolved interpolation. In parallel, simplifying input requirements and enhancing the generalizability of these methods remain key goals, particularly in settings where computational resources or system-specific information are limited. Therefore, there remains a pressing need for MSM frameworks that operate on low-dimensional, system-agnostic observables and are readily applicable to small, driven, and heterogeneous systems.

The stochastic landscape method (SLM)^73^ offers a physically grounded approach to coarse-graining dynamic self-assembly processes. The SLM builds on principles from transition state theory^74^ and leverages the relationship between structure and fluctuations^75^ to extract kinetic information directly from energy trajectories by identifying changes in dynamical trends.^76^ This framework has been shown to accurately predict nonequilibrium assembly times,^73^ facilitate the control of drive activation protocols,^11^ and classify metastable configurations in protein folding landscapes.^77^ While conventional MSM construction often relies on high-dimensional data, the SLM operates on a single observable, remains sensitive to stochastic effects in small systems, and does not require exhaustive sampling. These features make it a promising foundation for building minimal, interpretable Markov state models tailored to nonequilibrium self-assembly.

In our recent SLM work,^11^ we divided energy trajectories into segments using the BEAST (Bayesian Estimator of Abrupt Changes, Seasonality, and Trend) trendspotting algorithm and summarized each segment by its mean energy and mean trend (named the stochastic coordinates) and dwell times. From these summaries, we identified a near-zero-trend trap region for segments exhibiting long dwell times, i.e., metastable segments. Utilizing this result, we implemented a closed-loop “shock” that briefly weakens interactions only when the system resides in that region. In strong-binding regimes, this targeted actuation significantly increased assembly yield and shortened the time-to-first-assembly compared with the undriven baseline, establishing SLM as a practical, data-light method to diagnose and relieve kinetic trapping.

Here, we use SLM-derived observables to construct minimal MSM surrogates, using the same KMC model and the closed-loop activation scheme from our earlier work.^11^ The SLM-derived features, specifically, the mean energy and mean trend of segmented trajectories, are utilized to define physically informed state discretizations and transition kinetics, resulting in a minimal and interpretable Markov model of the assembly process. Using relatively modest simulation data, we show that the SLM-based MSM quantitatively reproduces the assembly yields and first-assembly time distributions for two equilibrium test cases. We further demonstrate that by combining the two equilibrium MSMs with a driven shock activation protocol, a nonequilibrium MSM can be reconstructed. Within this framework, kinetic traps are identified through near-zero trends in the energy landscape, enabling targeted activation to facilitate escape and promote assembly (see Figure 1). The resulting driven MSM successfully captures the dynamics of nonequilibrium self-assembly and, importantly, generalizes to previously unexplored physical parameter regimes while reducing computational costs by several orders of magnitude. Finally, we show that the model provides a practical tool for comparing and optimizing alternative driving protocols through the minimization of task-specific cost functions, with predictions validated against full-scale simulations. While demonstrated here for lattice-based nonequilibrium self-assembly, the framework is broadly applicable to other many-body systems exhibiting stochastic dynamics.

**Figure 1:**
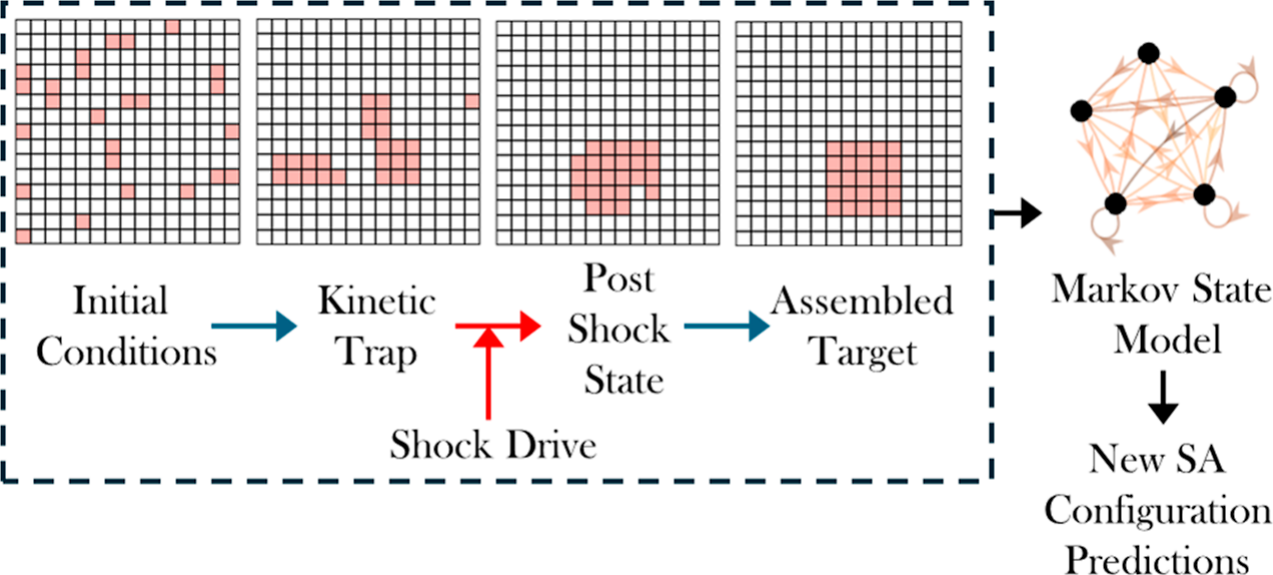
Overview of the proposed method. The self-assembly (SA) process initiates from random initial conditions and evolves until the system becomes kinetically trapped. At this point, a nonequilibrium shock is conditionally applied to modify the interaction energies and promote escape from traps. The resulting dynamics are coarse-grained into a Markov state model, providing an interpretable and computationally efficient framework that enables predictive insights beyond the original training set.

## Model

### Self Assembly Model

We simulate a kinetic Monte Carlo (KMC) model of self-assembling particles on a two-dimensional lattice, balancing computational tractability with biologically inspired complexity, following our previous work.^11^ The particles interact with their nearest neighbors via pairwise energies determined by their internal states and the encoded target structures. These interactions can be either strong (*J*_s_) or weak (*J*_w_), where |*J*_s_| > |*J*_w_|. The heterogeneity of binding energies encodes the design of the target structures and introduces energetic frustration into the assembly process. At each KMC step, particles can undergo either a spatial move or an internal state transition, with rates governed by local energy differences (see Figure S1 in the Supporting Information).

Under equilibrium conditions, the dynamics satisfy detailed balance, with transition probabilities determined by Boltzmann-weighted energy changes. This enables efficient exploration of the system’s energy landscape while enforcing steric and bonding constraints. The KMC transition rates are system-dependent. Here we adopt the form of Section S1 of the Supporting Information. This choice is physically motivated: we set state-switching and lattice-translation moves to occur on comparable time scales, with the translational move prefactor scaled to the diffusion time scale of a protein of its own size. Boltzmann factors are included symmetrically in a minimal form that enforces detailed balance.

To simulate nonequilibrium conditions, we employ a real-time, closed-loop control scheme based on dynamical trend analysis, which modifies the interaction energies to promote escape from kinetic traps. This approach follows the strategy outlined in our previous work.^11^ The KMC simulation rules (Figures S1A–C), equilibrium realizations examples (Figures S1D and S2), default parameters (Table S1), and rate constant calculation are described in full in Section S1 of the Supporting Information.

For this study, we chose to construct MSMs based on two representative configurations under equilibrium conditions and one under driven, nonequilibrium conditions. These baseline scenarios were selected to systematically demonstrate the method’s ability to capture transitions induced by external driving. In particular, a nonequilibrium drive implemented by scaling interaction energies, mimicking, for instance, the effect of pH changes^11,78^ or laser heating,^70^ can be interpreted as the weaving of two distinct equilibrium energy landscapes and the transitions between them. This perspective offers physical intuition for the effectiveness of such driving protocols, rendering energy scaling as a principled mechanism for steering self-assembly processes.

### Equilibrium SLM Data Set

To construct the equilibrium MSMs, we first generate the corresponding SLM equilibrium data sets, corresponding to two distinct energy configurations. These data sets are compiled from ensembles of KMC simulation trajectories, following the methodology established in our previous work.^11,73,77^

The KMC trajectories are recorded as continuous time series of the total system energy, which we use to construct the stochastic landscape coordinates. We select energy as the reaction coordinate due to its direct link to barrier crossing in transition-state theory. Still, the SLM is general and can be applied to other observables, including geometric measures that capture metastable states, such as solvent-accessible surface area (SASA) and dispersion degree,^79^ as well as spin-based coarse variables (e.g., principal components of spin configurations) used to detect phase transitions.^80^ In our lattice model, energy trajectories are highly correlated with these geometric descriptors (see Supporting Information Section S1 and Figure S3), supporting energy as a reliable surrogate for detecting metastable states. While distinct configurations may share similar energies, the SLM segments trajectories by both energy and its short-time trend, under the assumption that states with comparable values of these stochastic coordinates also exhibit similar kinetic behavior, even if they are clumped together in the coarse space. More broadly, the SLM is agnostic to the observable and can be extended to an integrated, weighted variable that combines multiple descriptors.

Nevertheless, energy can under-resolve metastability in entropy-dominated assemblies (e.g., hard-sphere-like), where structure changes with minimal variation in potential energy.^28,81^ In larger systems, self-averaging further damps energy fluctuations, and distinct macrostates may be nearly energy-degenerate yet separated by entropic bottlenecks, yielding different kinetics despite similar energy traces.^82^ In such cases, we suggest simply replacing energy with a structural or data-driven slow coordinate while retaining the same 1-D SLM pipeline, for example, bond-orientational order or crystalline fraction,^68^ largest-cluster size,^79^ or a learned slow mode via tICA/VAC/VAMPnets.^83–85^

The parameters appearing in this section and subsequent ones are defined in Table S2 in Section S2 of the Supporting Information. We simulate *N*_*i*_^eq^ realizations of the equilibrium KMC dynamics for two distinct system configurations indexed by *i* = 1, 2, starting from random initial conditions, collectively referred to as the KMC simulation ensembles. The configuration *i* = 1 employs the default simulation parameters, including the interaction energies *J*_s_ and *J*_w_ (see Table S1 in the Supporting Information). Configuration *i* = 2 is identical except for reduced interaction energies, scaled by a factor ρ > 1, namely *J*_s_/ρ and *J*_w_/ρ. The corresponding equilibrium SLM data sets are denoted by Ω_*i*_^eq^.

For each simulation trajectory of the two KMC simulation ensembles, we analyze the time evolution of the system in real time. The trajectory is segmented into consecutive subtrajectories, to be analyzed using BEAST for trend change-points detection. The duration of each subtrajectory, which contains the energy values and corresponding times generated by the self-assembly model, is set to τ_BEAST,*i*_, defined separately for each configuration *i* = 1, 2, as described in Section S1 of the Supporting Information. A real-time data extraction process is applied to each subtrajectory for the KMC equilibrium conditions (blue arrows in Figure 2A). The nonequilibrium data set extraction process (indicated by red arrows) is introduced in the following subsection.

**Figure 2:**
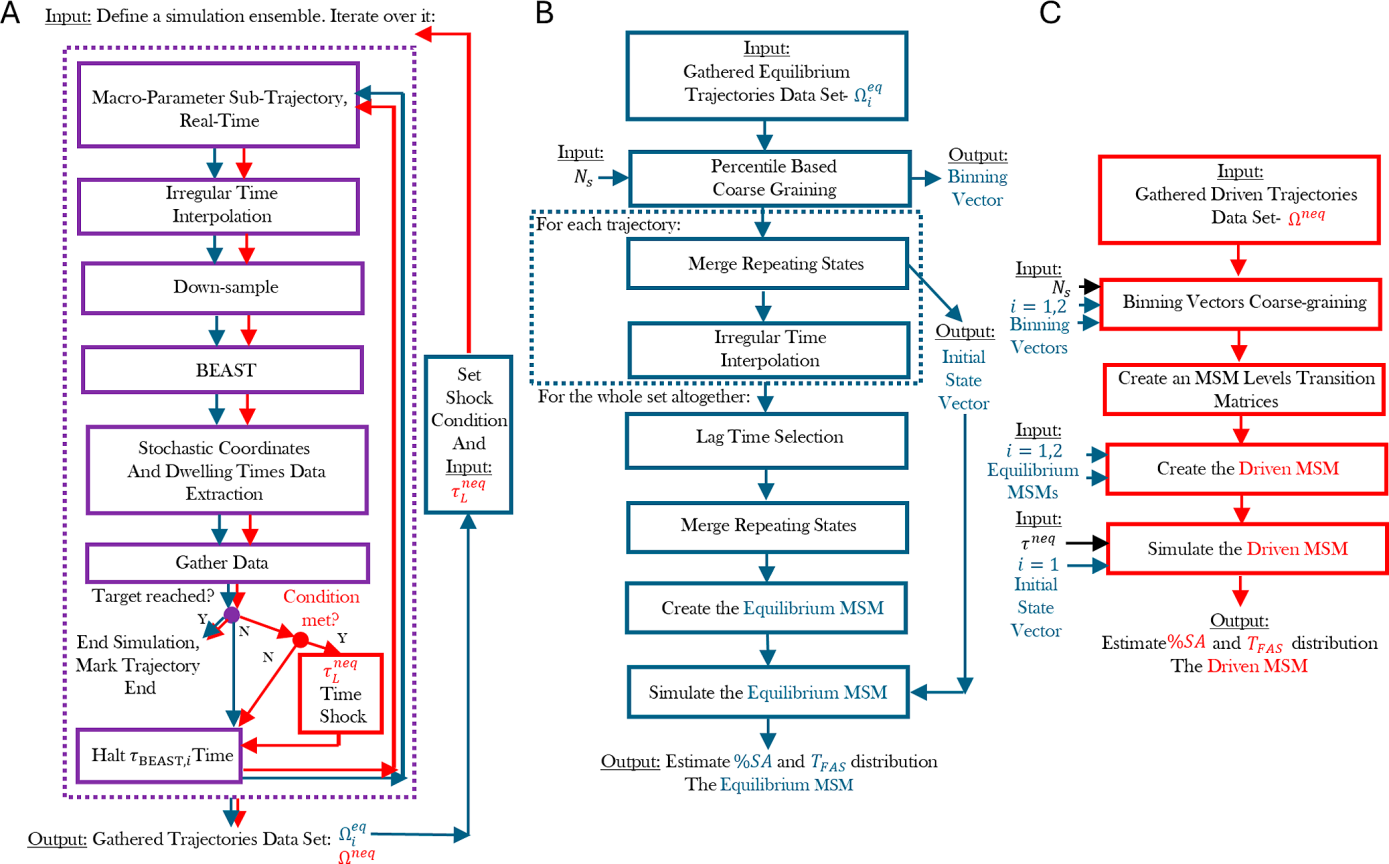
Coarse-graining self-assembly systems using the stochastic landscape method for the construction of Markov state models. (A) Schematic of the SLM-based nonequilibrium control protocol. Equilibrium simulations are first conducted for configurations *i* = 1, 2, yielding the data sets Ω_1_^eq^ and Ω_2_^eq^, which consist of stochastic coordinates (average energy and trend) and state dwell times up to the first assembly event (blue arrows). These equilibrium data sets are then used to define the drive activation condition for the nonequilibrium protocol, characterized by a drive duration τ_L_^neq^ and an energy down-scaling factor ρ, as described in the main text. A set of driven simulations is subsequently performed (red arrows), producing the nonequilibrium data set Ω^neq^. Blue and red arrows denote steps specific to equilibrium and driven conditions, respectively. (B) Construction of the equilibrium MSM using Ω_1_^eq^ and Ω_2_^eq^, and a predefined number of coarse-grained states *N*_s_. This process also defines the binning vectors and initial state distribution for configurations *i* = 1, 2, and yields the first assembly yield (% SA) and time-to-first-assembly *T*_FAS_ distribution. (C) Construction of the driven MSM by combining the two equilibrium *N*_s_-state MSMs for configurations *i* = 1 (predrive) and *i* = 2 (postdrive), along with their corresponding binning vectors. The initial conditions are based on the equilibrium distribution of configuration 1. The driven MSM is then used to estimate the assembly yield and the assembly time distribution under nonequilibrium conditions.

For equilibrium, each subtrajectory is first interpolated onto a uniform time grid with corresponding energy values. It is then downsampled by a factor of *d*_s_ to reduce computational demands. Next, BEAST algorithm^76^ is applied, assuming linear trends for change-point detection without seasonal components. The resulting segmentation defines intervals between consecutive trend changes. For each segment, we extract the stochastic coordinates, namely, the mean energy ⟨E*⟩ and the mean trend ⟨t*⟩, and record the corresponding number of KMC steps and dwell time *T*_dwell_, along with the onset and end times. These data are stored in Ω_*i*_^eq^.

At each segment, we evaluate whether the target structure has assembled. If assembly occurs, the simulation for that trajectory terminates, and all subsequent segments are excluded from Ω_*i*_^eq^. The first-assembly time *T*_FAS_ is recorded, along with an assembly flag set to 1. If the target structure is not assembled within the subtrajectory, the simulation advances by τ_BEAST,*i*_, generating the next subtrajectory, which is processed using the same scheme. This process repeats until assembly occurs or a maximum of *N*_steps_ KMC steps is reached. In the latter case, *T*_FAS_ is recorded as the final simulation time, and the assembly flag is set to 0, indicating no assembly has occurred.

This procedure is repeated for all trajectories in the KMC simulation ensemble. Once all segments have been processed, the final equilibrium data set output Ω_*i*_^eq^ is assembled and used for subsequent MSM construction.

### Nonequilibrium SLM Data Set

To construct the driven MSM, we generate the nonequilibrium SLM data set, denoted as Ω^neq^, using the SLM protocol. The first step involves defining the drive activation condition, following the approach introduced in our previous work.^11^ Using the equilibrium data set Ω_1_^eq^, generated with the default simulation parameters listed in Table S1, we identify a trap region T* centered around a zero mean trend (⟨t*⟩ = 0). This is done by sorting the segment-wise mean trend values, computing the smallest difference between consecutive values (the jump size), and expanding symmetric boundaries outward from zero in increments of the jump size. Expansion continues until the number of points within the interval exceeds $\left|\Omega_{1}^{eq}\right|/n_{bins}$ , where $\left|\Omega_{1}^{eq}\right|$ is the total number of segments in the data set Ω_1_^eq^ and *n*_bins_ is a chosen discretization parameter. The final interval defines T*, such that any mean trend value ⟨t*⟩ within these bounds is classified as part of the trap region.

This definition of the trap region enables the method to heuristically flag traps on the first encounter with a previously unvisited local minimum, even when that basin is absent from the learning set. This is especially useful in rugged energy landscapes, where many local minima share near-zero-trend signatures, allowing generalization to rare or sparsely sampled metastable states.

The drive activation condition applies whenever the current trend value falls within the trap region T*, triggering a shock that reduces the magnitudes of the interaction energies by a factor of ρ > 1 for a duration of τ_L_^neq^ seconds, where the subscript *L* denotes a learning process, as explained later in the text.

We emphasize that these nonequilibrium resets are applied by a selective feedback activation in the coarse-grained state space, which could potentially be implemented as a pH change^86^ or laser heating,^70^ to modulate the bond strength. Importantly, our framework is actuator-agnostic and aligns with the theory that informed resets accelerate search/assembly.^87–89^ We define the nonequilibrium simulation ensemble to consist of *N* ^neq^ realizations, each controlled in real time using a closed-loop feedback scheme (red arrows in Figure 2A). All simulations begin at configuration *i* = 1 with random initial conditions.

Each realization in the driven simulation ensemble follows a similar procedure as in the equilibrium case, illustrated in the dotted box of Figure 2A. The process begins with a subtrajectory over the interval *t* = 0 to τ_BEAST,*i*_, which is interpolated, downsampled by a factor *d*_s_, and segmented using the BEAST algorithm. For each segment, the mean energy, mean trend, dwell time, KMC number of steps, and onset and offset timestamps are recorded in Ω^neq^. If the target assembles within the current interval, the simulation ends, *T*_FAS_ is recorded, the assembly flag is set to 1, and no further segments are added for that trajectory. If not, the system checks the drive activation condition: when the current trend falls within the trap region T*, a shock is applied, reducing the interaction energies for τ_L_^neq^ seconds. After a delay of τ_BEAST,*i*_, the next subtrajectory begins from the end point of the previous one. If a shock was applied, its effect spans τ_L_^neq^ seconds within the new subtrajectory, with τ_L_^neq^ < τ_BEAST,1_ ensuring the system returns to baseline interaction energies before the next subtrajectory evaluation.

An example of this protocol is shown in Figure 3, illustrating energy fluctuations, trend segmentation, drive activation, and system snapshots highlighting key states. This data-gathering and drive–activation cycle repeats for each trajectory until assembly occurs or the maximum of *N*_steps_ KMC steps is reached. After processing all trajectories, the nonequilibrium data set Ω^neq^ is complete and ready for driven MSM construction.

**Figure 3:**
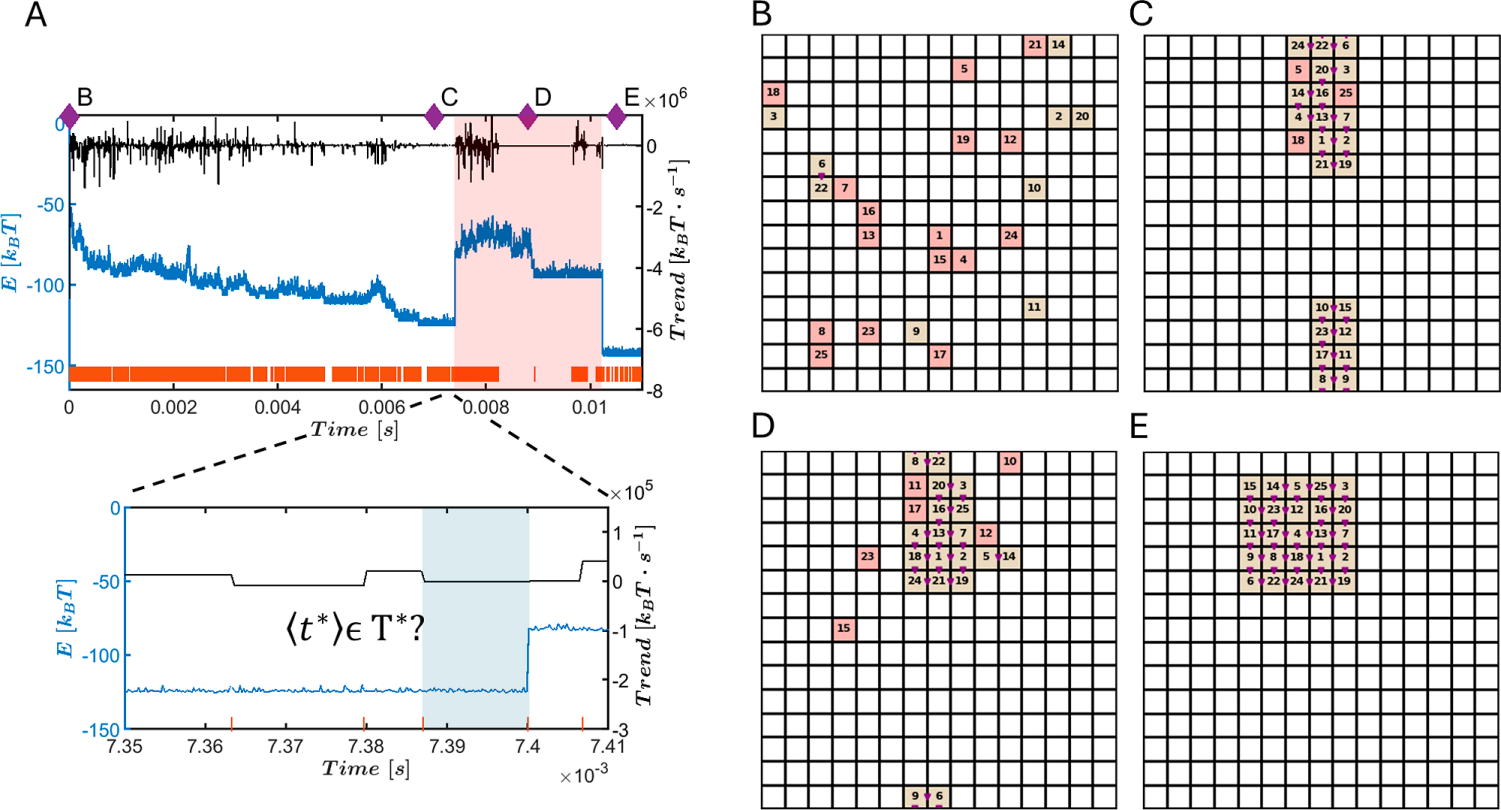
Driven self-assembly model. (A) Representative energy trajectory from a driven self-assembly simulation (blue, left *y*-axis). The system begins in equilibrium configuration *i* = 1 with strong and weak binding energies *J*_s_ = −3.6 [*k*_B_ *T*], *J*_w_ = −1 [*k*_B_ *T*], respectively, and is conditionally driven to configuration *i* = 2 with *J*_s_ = −2.4 [*k*_B_ *T*] and *J*_w_ = −0.67 [*k*_B_ *T*]. Energy segmentation is performed in real time using the BEAST algorithm, which evaluates the energy trend every τ_BEAST,*i*_ seconds. Segment boundaries are marked by orange vertical lines, and the corresponding segment trend values ⟨t*⟩ are plotted in black (right *y*-axis). Drive activation follows a closed-loop protocol, triggered only when the mean segment trend ⟨t*⟩ falls within the predefined trap region T* at the end of each τ_BEAST,1_ time interval, as described in the main text. A zoomed-in view of the drive activation window is shown in the lower blue-shaded panel, and the corresponding drive-activated energy segment is shaded in red in the main trajectory. Panels (B–E) display simulation snapshots corresponding to time points indicated by purple diamonds in (A), capturing key stages in the assembly process of the number-labeled distinguishable particles: (B) initial disordered configuration, (C) kinetic trap, (D) progression toward ordered structure, and (E) successful assembly of one of the stored target structures. In the snapshots, red and brown colors denote different particle internal-states. Strong interparticle bonds are represented by purple triangles and blue circles connecting adjacent particles.

### Constructing the Equilibrium MSM

The construction of the MSMs for the two equilibrium configurations successively follows the blue arrows in Figure 2B. The input includes the data set Ω_1_^eq^ and Ω_2_^eq^, which correspond to equilibrium simulations with the original and scaled energy values, respectively, as well as *N*_s_, the chosen number of coarse-grained states in the MSM. The adequacy of this coarse-graining approach in capturing dynamics of the underlying self-assembly system is assessed by comparing the MSM-derived results with the corresponding ground truth data. This validation strategy, based on output similarity aligns with the methodology introduced by Trubiano and Hagan,^42^ which we refer to as MSM results-based validation.

To construct the MSM, we analyze the Ω_*i*_^eq^ trajectory segments, alongside their associated *T*_FAS_ values and assembly flags, indicating whether assembly occurred. In formulating a coarse-graining strategy, we propose the following hypothesis: segments with similar values of the stochastic coordinates, namely, the mean energy and the mean trend, are likely to be physically similar. Accordingly, we group segments with sufficiently close values of these two observables into the same coarse-grained state, each represented by a characteristic ordered pair of energy and trend.

To coarse-grain the system stochastic behavior while avoiding an excessive number of states, we introduce an additional guiding hypothesis: states can be considered similar if their stochastic coordinate values fall within similar quantiles of the empirical distributions of energy or trend, measured over the entire data set of trajectory segments. This hypothesis is motivated by the observation that complex many-body systems often exhibit qualitatively different kinetic behavior across time scales. In particular, long-time dynamics are frequently dominated by kinetic traps that lead to slow relaxation and large fluctuations in stochastic observables.^90,91^ In our prior work,^11^ we observed that the trend values varied by orders of magnitude depending on whether the system was kinetically trapped or not. Consequently, applying a fixed bin width for coarse-graining can be problematic, as large bins may obscure kinetically distinct behaviors by merging dissimilar segments, while narrow bins may fragment the data into too many states, compromising the statistical robustness of the resulting MSM. To address this, we introduce a quantile-based coarse-graining scheme, described in Subsection S3.1 of the Supporting Information. This adaptive binning method partitions the data according to the empirical distributions of the trend and energy values, enabling us to retain important kinetic distinctions while keeping the number of coarse-grained states manageable. The resulting scheme assigns each trajectory segment to a labeled and numbered coarse-grained state and also generates a binning vector that is later reused for constructing the MSM under driven conditions.

The coarse-grained data set is then partitioned into trajectory subsets based on the onset times of the segments. Each subset corresponds to a single KMC trajectory and is identified by the onset time of its first segment (always 0 s, consistent with the original simulation) and the final segment’s end time. For each subset, we extract the sequence of state numbers, ordered by segment onset time. To construct a continuous-time Markov state model (MSM),^92,93^ we merge consecutive segments with identical state numbers into a single dwell period. The onset time of each merged segment is taken from the first occurrence in the sequence, and its dwell time is computed as the total duration of the merged segments. Each resulting trajectory, consisting of merged coarse-grained state sequences, is then interpolated onto a uniform time grid. The time step for this grid is set to 50 times the smallest time difference between consecutive segment onset times in the original trajectory. This step balances memory efficiency with the accuracy of the interpolated representation. Note that this uniform resampling process introduces repeated state numbers and removes the original continuous-time structure. This limitation is addressed and corrected in subsequent steps of the analysis.

We iterate over all updated trajectory subsets to collect the state number of each subset’s first segment. The number of occurrences of these initial states are then normalized by the total number of trajectories, yielding the initial distribution over coarse-grained states. This distribution is stored as the initial state vector for the corresponding equilibrium configuration.

Next, we apply a lag time selection procedure^42,47^ to the subset data. This involves downsampling the state-number-versus-time vectors at a selected lag time while preserving their discrete sequence structure on a uniform time grid. As in prior studies,^47,59,60,94^ this step mitigates short-term memory effects. Rather than relying solely on common criteria for choosing lag time, such as minimizing Chapman–Kolmogorov error or according to the time scale associated with the transition matrix, we seek to balance two competing objectives: (i) suppressing memory effects, and (ii) retaining sufficient temporal resolution to capture early assembly dynamics. To ensure the model can resolve the shortest observed first assembly time (*T*_FAS_) in Ω_*i*_^eq^, we choose the lag time so that even the shortest trajectory includes at least five state transitions.

Nevertheless, our lag time choice is not intended to enforce Markovianity. Requiring at least five samples per trajectory to capture the fastest assembly events precludes a memoryless description in our system. To show this, we analyze the system implied time scales and conduct the Chapman–Kolmogorov test.^94^

The subsets are then downsampled according to the lag time selection, with the beginning of each time series trimmed as needed to align with the lag intervals. In each updated Ω_*i*_^eq^ subset, adjacent segments assigned to the same state are merged again. The resulting data set is then used to construct a continuous-time state model by computing the state transition matrix and characteristic dwell time vector, as detailed in Subsection 3.2 of the Supporting Information. With these components, the transition matrix, coarse-grained state definitions, and dwell time vector, the state model is represented as a weighted, directed graph.

This yields a Markovian surrogate (MSM) by design: from the coarse-grained (non-Markovian) dynamics at the chosen lag, we retain the same state labels, state transitions, and assign each state an exponential dwell-time distribution whose mean matches its empirical dwell time; the resulting continuous time Markov chain over the graph defines the MSM. We then assess the adequacy of this Markovian approximation in a results-oriented manner, similar to Trubiano and Hagan’s work.^42^ Rather than reproducing every microscopic feature of the KMC, we test whether the MSM recovers the emergent observables of interest (yield and the first-assembly time distribution). When these targets are reproduced to acceptable accuracy, the neglect of memory is justified for our purposes.

Each MSM is then simulated following the procedure detailed in Subsection 3.3 of the Supporting Information. From the full ensemble of simulated trajectories, we compute the predicted assembly yield (% SA) and *T*_FAS_ distribution using Kernel density estimation.^95^ These are compared to the corresponding ground truth values to assess the MSM’s accuracy in reproducing the original assembly dynamics.

### Constructing the Driven MSM

To construct the driven Markov state model (MSM), the following inputs are required: the number of coarse-grained states *N*_s_, the data set of nonequilibrium (driven) trajectories Ω^neq^, the shock duration τ_L_^neq^, the subtrajectory interval duration τ_BEAST,*i*_ and the equilibrium MSMs for configurations *i* = 1 and 2, and their associated binning schemes. The initial state distribution of the driven MSM is taken from the initial state vector of the equilibrium MSM for configuration *i* = 1.

The data set Ω^neq^ comprises trajectories in which the system alternates between configurations 1 and 2 due to repeated drive activation and deactivation. These data are used to analyze transitions from configuration 1 to 2 upon drive onset, and from 2 to 1 upon drive termination. We assume that the system’s behavior between these transitions is adequately captured by the corresponding equilibrium MSMs of configurations 1 and 2. To coarse-grain Ω^neq^, we divide the trajectories into subsets, analogous to the approach used for the equilibrium data. Segment states during the drive-on periods are mapped using the binning scheme of MSM of configuration 2, while segments during drive-off periods are mapped using that of the MSM of configuration 1. Next, we identify the time points at which each trajectory transitions between drive-on and drive-off conditions. Using these points, we construct two *N*_s_ × *N*_s_ matrices: the forward level-jumping matrix (*T*_F_), representing transitions from configuration 1 to 2 upon drive activation, and the backward level-jumping matrix (*T*_B_), representing transitions from configuration 2 to 1 upon drive deactivation. The construction of *T*_F_ and *T*_B_ is described in detail in Subsection S3.4 of Supporting Information.

This procedure produces a two-layer Markov model, referred to as the driven MSM. The first layer corresponds to the equilibrium regime of configuration *i* = 1, and the second to that of configuration *i* = 2. Transitions between layers follow a condition that mirrors the drive activation scheme shown in Figure 2A. The data set Ω^neq^ was constructed using drive activations of fixed duration τ_L_^neq^, hence the subscript *L* is used to distinguish this specific value from other tested durations of τ^neq^. These additional durations were used to evaluate the model’s generalization, and were not included in training or tuning.

Simulation of the driven MSM follows the procedure outlined in Subsection S4.5 of the Supporting Information, yielding predictions for the assembly yield (% SA) and *T*_FAS_ distribution. In addition, for the nonequilibrium case, we evaluate a third metric: the total drive activation time. This is computed by summing all active drive interval KMC steps within each simulation realization. Then, *T*_Drive_ is defined as the ensemble mean of this summed value. This metric is particularly relevant, as extended activation in real systems carries physical and energetic costs.

## Results and Discussion

### Ground Truth Results Collection

To construct the MSM, we employ the stochastic landscape method, a data-driven coarse-graining approach that segments a one-dimensional energy observable into discrete states using trend-based change-point detection. From kinetic Monte Carlo simulated as continuous-time Markov processes, we generate three key data sets: Ω_1_^eq^, which contain equilibrium trajectories with default binding energy values (Table S1), referred to as configuration 1, Ω_2_^eq^, which contain equilibrium trajectories with binding energy values scaled down by a factor of ρ = 1.5, referred to as configuration 2, and Ω^neq^, which consists of trajectories that alternate between these configurations under drive activation and deactivation. Equilibrium MSMs are independently constructed for each configuration using downsampled data, and a driven MSM is built by connecting them through level-jumping matrices informed by Ω^neq^.

To assess the MSM ability to capture self-assembly dynamics under both equilibrium and nonequilibrium conditions, we compare its predictions for assembly yield (% SA), defined as the percentage of simulations that successfully reached the target structure within the predefined number of KMC steps *N*_steps_, and the distribution of first assembly times (*T*_FAS_) to the corresponding outcomes from KMC simulations. For each KMC ensemble used to construct Ω_1_^eq^, Ω_2_^eq^, and Ω^neq^, we extract empirical *T*_FAS_ values and compute their distributions using histograms and kernel density estimation (KDE).

To evaluate the generalization performance of the MSM under nonequilibrium conditions, we simulate a range of τ^neq^ values, 0.034·τ_BEAST,1_ ≤ τ^neq^ ≤ 0.77·τ_BEAST,1_, following the red arrows in Figure 2A. For this purpose, we consider only the resulting *T*_FAS_ distributions and yield values, and do not retain the generated Ω^neq^ data sets. The resulting yield curve as a function of τ^neq^ is shown in Figure S4 and was used to guide the selection of τ_L_^neq^, as explained in Section S2.4 in the Supporting Information.

### Simulation Details

Here we outline the simulation setup and parameter values used to generate the equilibrium and driven trajectory data sets, Ω_1_^eq^, Ω_2_^eq^, and Ω^neq^, as well as the construction and validation of the corresponding MSMs. We describe the energy parameters defining each configuration, the KMC simulation conditions, and the statistical settings used for model training and evaluation.

KMC simulations for configuration 1 were performed using the parameters listed in Table S1, with interaction strengths set to *J*_s_ = −3.6 [*k*_B_ *T*] and *J*_w_ = −1 [*k*_B_ *T*]. Configuration 2 shares the same parameter table but uses weaker interactions, scaled by ρ = 1.5, namely *J*_s_ = −2.4 [*k*_B_ *T*] and *J*_w_ = −0.67 [*k*_B_ *T*]. The equilibrium ensembles comprise *N*_1_^eq^ = 300 and *N*_2_^eq^ = 60 simulations for configurations 1 and 2, respectively. These follow the equilibrium simulation protocol shown in Figure 2A, with a downsampling index of *d*_s_ = 100 and BEAST activation windows of τ_BEAST,1_ = 3.7 × 10^–3^ s and τ_BEAST,2_ = 0.54 × 10^–3^ s, producing the data sets Ω_1_^eq^ and Ω_2_^eq^. The rationale behind all parameter choices, including *N*_1_^eq^, *N*_2_^eq^, τ_BEAST,1_, τ_BEAST,2_, τ_L_^neq^, the equilibrium MSM transition matrices, and the jump matrices *T*_F_ and *T*_B_, is detailed in Section S2 of the Supporting Information; Section S2 of the Supporting Information additionally reports a one-factor-at-a-time sensitivity analysis of the BEAST parameters (window, downsampling, noise), with quantitative overlap metrics in Figure S5. The distributions of ⟨t*⟩ and ⟨E*⟩ for the same trajectory are stable to halving/doubling τ_BEAST,1_, changing the downsample index. Considerable degradation occurs only under injected additive white Gaussian noise (5 dB signal-to-noise ratio (SNR)) and near-baseline overlap at 20 dB SNR.

We now examine the memory feature of the KMC system. As shown in the Supporting Information Section S4.1 for configuration 1, at the chosen lag time, the implied time scales do not plateau (see Figure S6), and the return probabilities for two exemplar states deviate from empirical uncertainty, indicating non-Markovian behavior as per the Chapman–Kolmogorov test. Increasing the lag does not make the process Markovian. Still, it brings Chapman–Kolmogorov curves closer to their uncertainty bands^94^ and produces more pronounced implied time scales plateaus, at the cost of short-time resolution.

The condition for activating the drive in the nonequilibrium simulations is the current trend being within the trap region T*, defined based on Ω_1_^eq^ (see Figure S7 of the Supporting Information). The driven KMC ensemble includes *N* ^neq^ = 420 simulations following the protocol shown in Figure 2C, with a fixed drive period of τ_L_^neq^ = 0.035·τ_BEAST,1_. The MSMs for configurations *i* = 1 and *i* = 2 are constructed as shown in Figure 2B, with *N*_s_ = 10 and *N*_s_ = 26 coarse-grained states, respectively. Each model includes the initial state vector, coarse-graining bin edges, dwell time distributions, and state labels. The resulting MSMs are then simulated to generate predictions of assembly yields (% SA) and first assembly time (*T*_FAS_) distributions, which are compared to the KMC ground truth. Next, using the outputs of the equilibrium MSMs together with τ_BEAST,1_, τ_L_^neq^, and *N*_s_, a driven MSM is constructed as shown in Figure 2C. Yield and *T*_FAS_ predictions from this model, for both *N*_s_ = 10 and *N*_s_ = 26, are compared to those from the corresponding KMC simulations.

Finally, the driven MSM is used to forecast yields, mean total drive activation duration, and first assembly time distributions across a range of τ^neq^ values for both *N*_s_ = 10 and *N*_s_ = 26. These results are compared with ground truth KMC simulations to assess accuracy. The cost function associated with each protocol is then computed and analyzed, enabling a quantitative comparison between model predictions and ground truth performance.

### Equilibrium MSMs Results

To validate the equilibrium coarse-graining strategy, we begin by analyzing the performance of the MSMs constructed from the equilibrium ensembles Ω_1_^eq^ and Ω_2_^eq^. These data sets contain KMC simulation outputs performed under two different energy configurations, namely, configuration 1 with default binding energy values (Table S1), and configuration 2 with reduced interaction energy values, which facilitates the escape from kinetic traps. Using these ensembles, MSMs were trained independently to model the system’s dynamics under steady, nondriven conditions. Here, we assess the fidelity of each equilibrium MSM in capturing both kinetic and structural features of the system through its coarse-grained dynamics.

The initial distributions of the coarse-grained states of the equilibrium MSMs are shown in Figure S8, and the coarse-grained state labels are listed in Table S3 for *N*_s_ = 10 and Table S4 for *N*_s_ = 26, respectively. The MSMs of configurations *i* = 1 and *i* = 2 are depicted in Figure 4A,B for *N*_s_ = 10, respectively, and in Figures S9 and S10 for *N*_s_ = 26, respectively. Since our MSM construction includes a sink, states in the MSM are not visually rescaled in any of the figures according to the stationary probability. Visual representation of states by illustrative system snapshots for the default configuration MSM, and their physical interpretations are given in Section S4 and Figure S11 of the Supporting Information.

**Figure 4:**
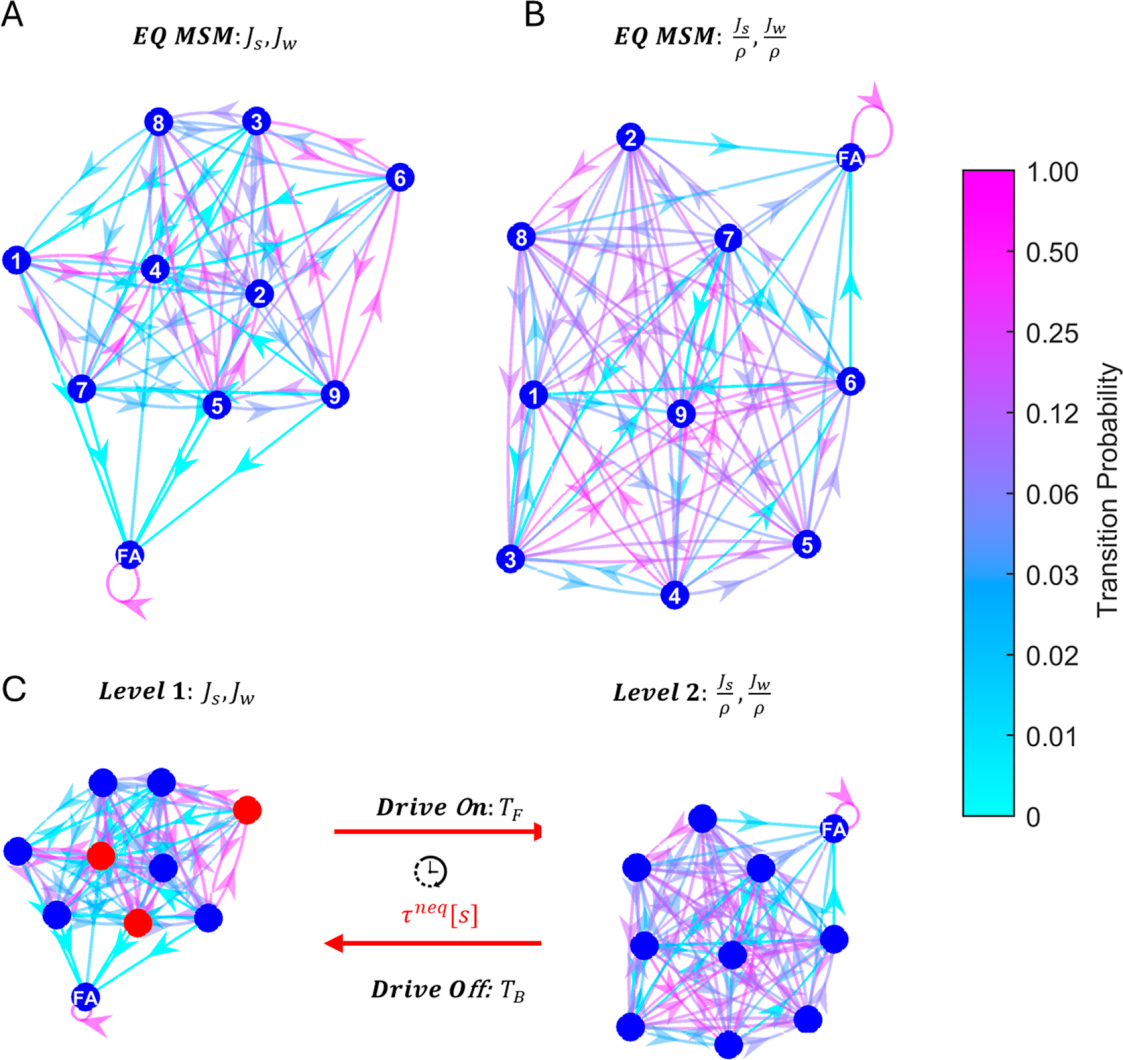
Equilibrium and driven Markov state models. Equilibrium Markov state model graphs of *N*_s_ = 10 states for configurations (A) *i* = 1 (before shock, corresponding equilibrium values) and (B) *i* = 2 (after shock, corresponding equilibrium values), respectively. Each node corresponds to a coarse-grained state defined by its mean energy and mean trend values, except for the absorbing state labeled FA, which represents successful first assembly. The state-specific energy and trend values are listed in Table S3 of the Supporting Information. Directed arrows indicate transitions between states, with arrow color denoting transition probabilities as specified by the color bar. (C) The driven MSM is schematically illustrated, constructed from the two equilibrium MSMs treated as distinct levels. Red nodes indicate states from which upward level transitions (drive activation) occur, as determined by the nonequilibrium control scheme described in the main text. Transitions between levels persist for a time interval τ^neq^. Red arrows represent level-jumping transitions: drive activation (forward jump) governed by the matrix *T*_F_, and drive deactivation (backward jump) governed by *T*_B_.

The dwell time distributions *T*_dwell_ for each MSM state are shown as histograms in Figures S12–S15. These are compared to fitted exponential distributions constructed to match the measured mean dwell times and binned using identical bin edges. For *N*_s_ = 10, distributions for configurations 1 and 2 appear in Figures S12 and S13, respectively, and the corresponding distributions for *N*_s_ = 26 are shown in Figures S14 and S15.

To quantify the agreement between empirical and exponential fits, we compute discrete Pearson correlation coefficients, *R*, for each *T*_dwell_ histogram comparison. The resulting correlation values range from *R* = 0.91 to *R* = 1.00 (rounded to two decimal places), demonstrating excellent agreement. These results suggest that the proposed coarse-graining procedure preserves a key aspect of Markovian (memoryless) behavior.^96^ Further insights into the model dynamics are provided in Figure S16, which shows the average dwell time ⟨*T*_dwell_⟩ for configuration 1 with *N*_s_ = 26. As expected, ⟨*T*_dwell_⟩ increases with the mean trend closer to zero and becomes even more pronounced at lower mean energy within the same trend bin, consistent with previous findings.^11^

Figure 5 presents the equilibrium *T*_FAS_ distributions generated by the MSMs alongside those obtained from the original KMC simulations, for configurations 1 (top row, panels A, B) and 2 (bottom row, panels C, D), and for *N*_s_ = 10 (left column) and *N*_s_ = 26 (right column) coarse-grained states, respectively.

**Figure 5:**
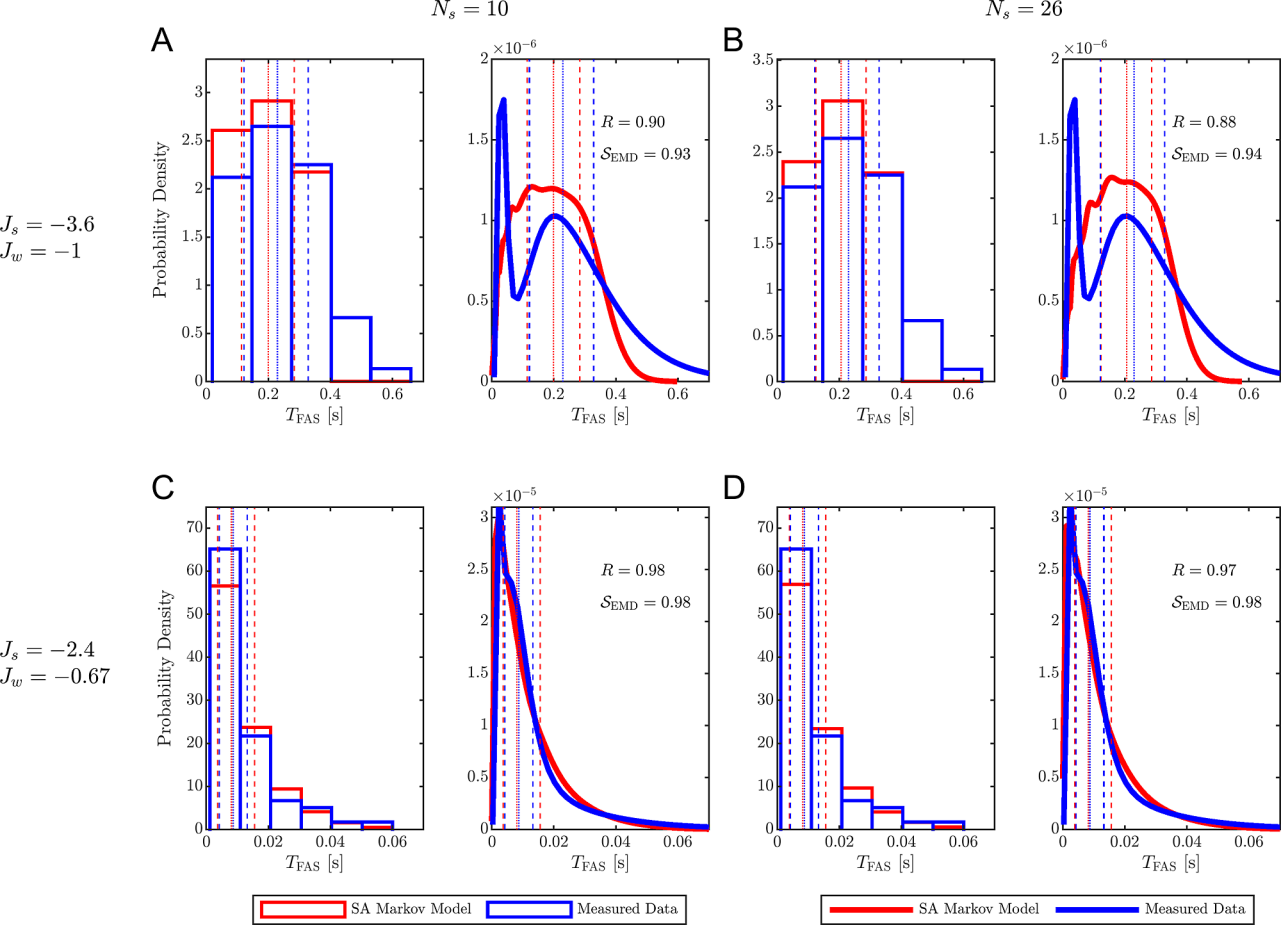
Comparison of equilibrium MSM predictions and KMC simulations for time-to-first-assembly distributions. (A) First assembly time *T*_FAS_ distribution for configuration 1 with *N*_s_ = 10 states and (B) *N*_s_ = 26 states, and for (C) configuration 2 with *N*_s_ = 10 states and (D) *N*_s_ = 26 states, comparing *T*_FAS_ predicted by the equilibrium Markov state model (red) with those obtained from direct kinetic Monte Carlo (KMC) simulations (blue). For each condition, the left plot displays histograms of *T*_FAS_ values, binned using Scott’s rule and normalized to yield probability densities. The right plot shows kernel density estimates of the same distributions. Vertical dashed lines mark the 25th and 75th percentiles, and the dotted line indicates the median (50th percentile) of each distribution. Pearson correlation coefficients *R* and the similarity measures *S*_EMD_ between the KDE curves are reported to quantify the agreement between MSM predictions and simulation data.

To compare the MSM and KMC distributions, we pair a familiar shape measure, Pearson’s correlation, with a transport-based metric, the 1-Wasserstein (Earth Mover’s) distance^97^

1 $$W_{1}=\int_{0}^{\infty }\left|F_{MSM}\left(\mathrm{t̃}\right)-F_{empirical}\left(\mathrm{t̃}\right)\right|d\mathrm{t̃}$$

where *F*_MSM_(*T*_FAS_) and *F*_empirical_(*T*_FAS_) denote the cumulative distribution functions of the estimated and measured *T*_FAS_ distributions, respectively. Pearson’s *R* is intuitive, but it can be fragile when differences live in heavy tails or under skew, whereas the Wasserstein view quantifies the minimal “work” required to morph one distribution into the other and remains informative when small amounts of probability mass are displaced far from the bulk. In one dimension, this distance equals the area between the two cumulative distribution functions. Practically, we compute the CDFs, measure the area between them over the bulk (up to the larger 99th percentile of either curve), normalize by the length of that interval to obtain a unitless quantity, and report a similarity *S*_EMD_ as one minus that normalized distance, similar to a previous normalized Wasserstein,^98^ and our normalization serves the same goal of robust comparability across curves. Together, *R* captures overall covariation in shape, and *S*_EMD_ reflects mass displacement, providing complementary evidence of agreement.

In all cases, MSM-predicted distributions align closely with the ground truth, both for the histogram and the KDE fit curves. The Pearson correlation between MSM and KMC KDE curves ranges from *R* = 0.88 to *R* = 0.98 and *S*_EMD_ from 0.93 to 0.94, indicating excellent agreement. Notably, configuration 2 shows the strongest agreement with *R* = 0.98, *S*_EMD_ = 0.98 for *N*_s_ = 10 and *R* = 0.97, *S*_EMD_ = 0.98 for *N*_s_ = 26, highlighting the MSM’s ability to capture equilibrium assembly kinetics with high accuracy.

In addition to kinetic profiles, the MSMs also reproduce assembly yields with high precision. For configuration 1, the KMC yield is 19.7%, while MSM-predicted yields are 17% and 16.5% for *N*_s_ = 10 and *N*_s_ = 26, respectively. For configuration 2, the yield reaches 100% in KMC simulations and is predicted as 99.7% and 99.5% by the MSM for *N*_s_ = 10 and *N*_s_ = 26, respectively. These results demonstrate the robustness of the MSM framework in capturing both first assembly kinetics and assembly yields.

### Validation of the Driven MSM on Training Data

We next turn to the nonequilibrium case and evaluate the driven MSM’s ability to reproduce the ground-truth dynamics used in its construction. The driven MSM is built using the nonequilibrium data set Ω^neq^, which consists of KMC trajectories postprocessed data alternating between equilibrium configurations 1 and 2 via repeated drive activation and deactivation. The drive protocol follows a trend-based triggering scheme, where transition events are determined by the trend value crossing a predefined threshold, with a fixed activation duration of τ_L_^neq^ in the learning phase. This training value enables consistent drive timing and allows construction of the forward and backward level-jumping matrices. The MSM-predicted observables are then compared against the corresponding ground-truth simulation data to assess how well the model captures the learned dynamics.

The driven MSM topology is illustrated schematically in Figure 4C for *N*_s_ = 10. A corresponding model is constructed for *N*_s_ = 26, where Figure S9 represents configuration 1 and Figure S10 represents configuration 2. These layers are connected via conditional transitions that mimic drive activation events occurring every τ_BEAST,1_ and lasting for a duration of τ^neq^. The coarse-grained states that trigger drive activation are highlighted as red nodes in Figures 4C and S9.

The corresponding forward and backward transition matrices, *T*_F_ and *T*_B_, for *N*_s_ = 10 and *N*_s_ = 26 are shown in Figure S17. As expected, *T*_F_ enables transitions from low-trend states to a range of target states following drive onset. In contrast, *T*_B_ displays only a weak dependence on trend during transitions from layer 2 to layer 1. The relevant state numbers and their respective values can be found in Table S3 and S4 of the Supporting Information.

Following the same validation strategy used for the equilibrium MSMs, we compare the driven MSM’s predictions for the yield and *T*_FAS_ distribution against the ground truth obtained from KMC simulations using τ_L_^neq^. These comparisons are presented in Figure 6A,B. For both values of *N*_s_, the predicted *T*_FAS_ distributions exhibit strong agreement with the KMC results, with Pearson correlation coefficients of *R* = 0.92 and *R* = 0.95 and *S*_EMD_ of 0.95 to 0.98, respectively. Yield predictions are also in good agreement. For KMC, the simulated yield is % SA = 47.7. The driven MSM predicts % SA = 46.4 for *N*_s_ = 10 and % SA = 33 for *N*_s_ = 26. These results indicate that the driven MSM effectively captures key aspects of the nonequilibrium dynamics, including both assembly timing and success probability.

**Figure 6:**
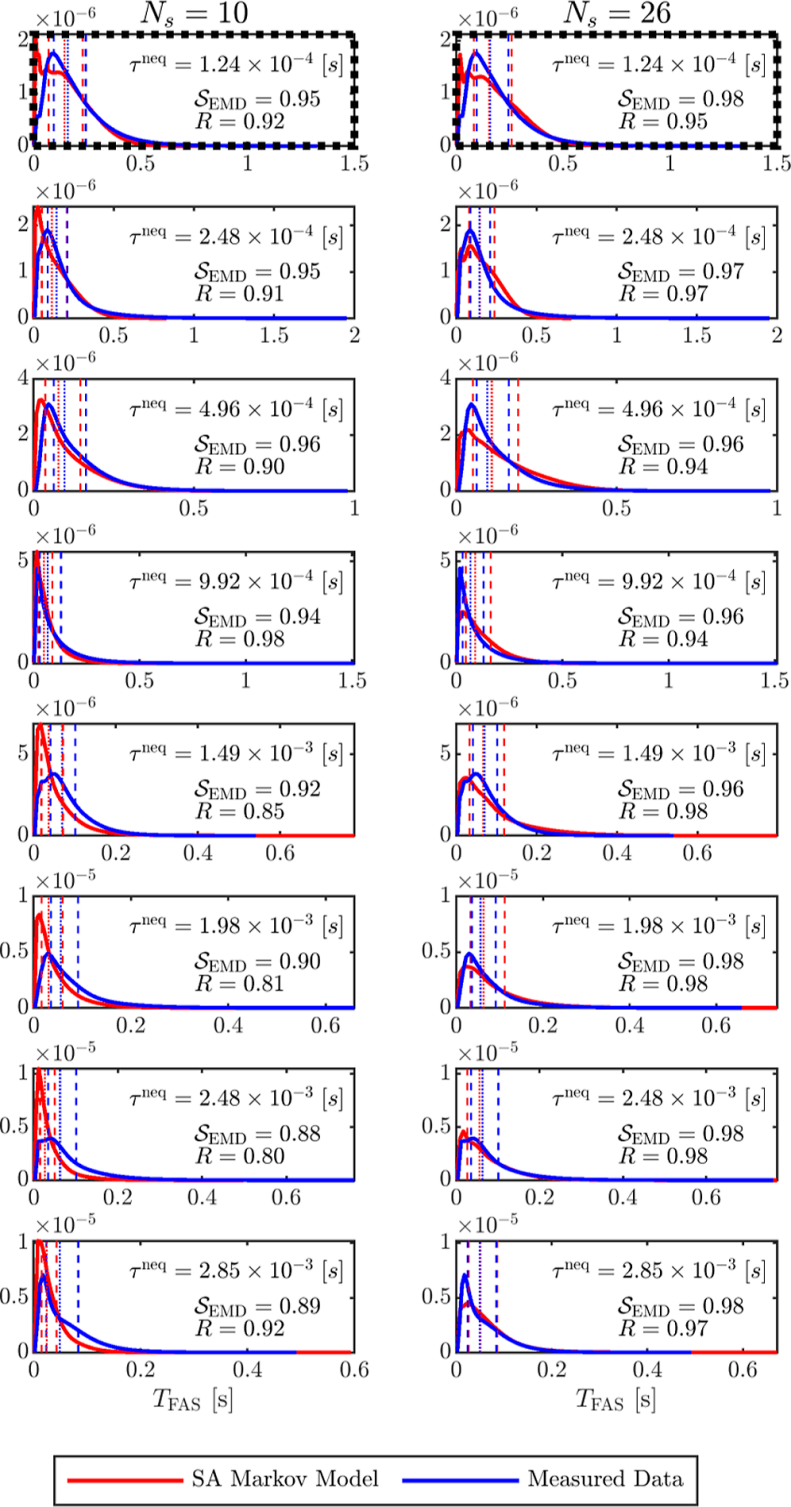
Comparison between MSM-predicted and KMC-simulated time-to-first-assembly distributions under driven conditions. The panels present the distributions of time to first assembly (*T*_FAS_) under varying drive activation window durations, τ^neq^, indicated in each subplot. Each row corresponds to a different value of τ^neq^, while the left and right columns show results for MSMs constructed with *N*_s_ = 10 and *N*_s_ = 26 coarse-grained states, respectively. In each panel, blue curves represent the distributions measured from kinetic Monte Carlo simulations, while red curves depict the corresponding predictions from the driven MSM. Vertical dashed lines mark the 25th and 75th percentiles, and the dotted line indicates the median of each distribution. The Pearson correlation coefficient *R* and the similarity measure *S*_EMD_, quantifying the agreement between the predicted and measured distributions, are shown in the bottom-right corner of each panel. Panels in the top row, outlined with dotted black borders, correspond to the drive duration τ^neq^ = τ_L_^neq^ used in the learning phase for constructing the level-jumping matrices as detailed in the main text.

### Generalization of the Driven MSM to New Drive Protocols

Having established that the driven MSM accurately reproduces its training data, we now investigate its generalization capability. Specifically, we simulate the driven MSM across a range of drive activation durations that were not used in training, and compare its predictions against full KMC simulations conducted under the same drive protocols. This evaluation focuses on key output observables, including the first assembly time distributions, assembly yields, and total drive activation times, and culminates in a cost function analysis that balances process efficiency and energy input. By doing so, we test the robustness of the MSM framework as a predictive tool and surrogate model beyond its training regime.

First, we evaluate the performance of the driven MSM beyond the training condition, specifically for various values of τ^neq^ different from the learning value τ_L_^neq^. The predicted *T*_FAS_ distributions from the MSM are compared to the corresponding KMC results in Figure 6, with each panel labeled by the simulated τ^neq^ value. Panels A and B display the baseline performance for τ^neq^ = τ_L_^neq^. Both *R* and *S*_EMD_ are computed for each distributions pair to quantify agreement. For all cases appearing in Figure 6, *S*_EMD_ is comparable to *R*, and thus we discuss only the Pearson coefficients.

For *N*_s_ = 10 coarse-grained states, the MSM predictions show strong agreement with KMC results for τ^neq^ ≤ 0.992 ms, with *R* values ranging from 0.90 to 0.98. At larger values of τ_neq_ = 1.49, 1.98, and 2.48 ms, the correlation slightly declines to *R* = 0.85, 0.81, and 0.85, respectively, indicating modest deviations. Notably, for τ^neq^ = 2.85 ms, the agreement improves again, with *R* = 0.92, suggesting a partial recovery in model accuracy at longer driving durations.

For *N*_s_ = 26, the driven MSM consistently achieves high correlation across the entire range of τ^neq^, with *R* values between 0.94 and 0.98. This robust performance is consistent with expectations, as a larger number of coarse-grained states better captures the fine-grained kinetic structure of the system. As discussed in Section S2.5 of the Supporting Information, both the MSMs equilibrium transition matrices and the forward-level jumping matrix, *T*_F_, were sufficiently sampled for each *N*_s_, supporting the improved accuracy at higher resolution.

The assembly yields as a function of τ^neq^ are presented in Figure 7A,B for both the MSM predictions and the ground truth data, while the corresponding mean total drive activation durations (*T*_Drive_) are shown in Figure 7C,D. For both *N*_s_ values, the MSM accurately reproduces the trends observed in the KMC simulations, demonstrating strong agreement in both yield and drive duration performance. As with the time-to-first-assembly *T*_FAS_ distributions, the predictive accuracy is higher for *N*_s_ = 26 than for *N*_s_ = 10, as the minimal difference between a pair of MSM-predicted and ground-truth values is smaller for *N*_s_ = 26 for both yield and *T*_Drive_, supporting the previous conclusion that a larger number of coarse-grained states better captures the system’s dynamics.

**Figure 7:**
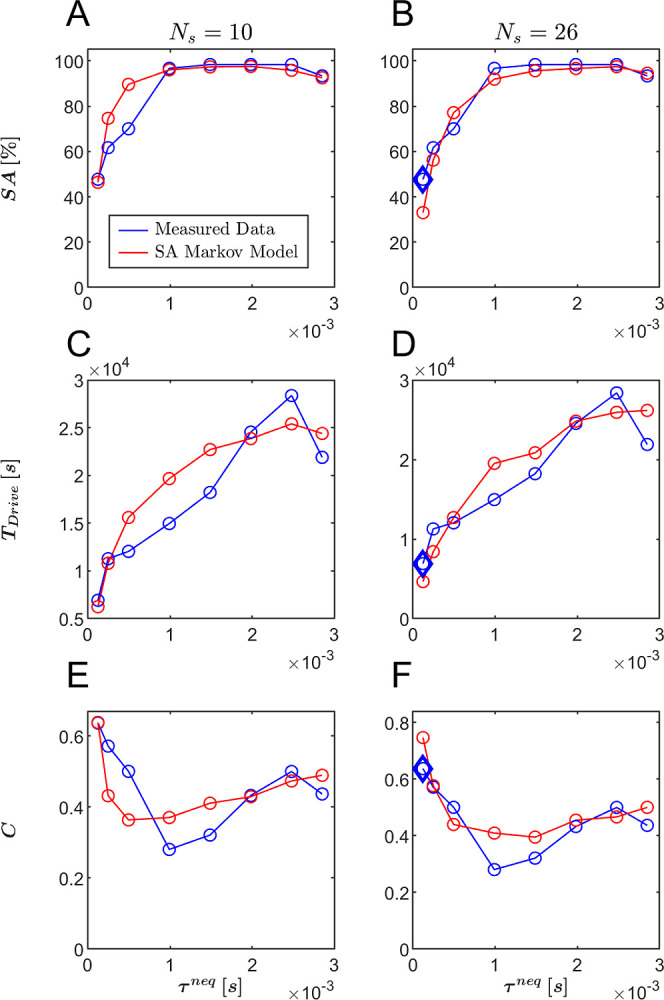
Quantitative comparison between KMC simulations (blue) and MSM predictions (red) across varying drive durations τ^neq^ of (A) Self-assembly yield, expressed as a percentage of successful assembly events % SA, with *N*_s_ = 10 states and (B) *N*_s_ = 26 states, (C) average total drive time required for each trajectory *T*_Drive_, with *N*_s_ = 10 states and (D) *N*_s_ = 26 states, (E) a composite cost function *C*(τ^neq^) combining normalized yield and drive time, used to evaluate trade-offs between assembly efficiency and resource expenditure associated with drive activations with *N*_s_ = 10 states and (F) *N*_s_ = 26 states. In all panels, diamond markers denote the specific value τ^neq^ = τ_L_^neq^ used in constructing the level-jumping transition matrices.

In realistic manufacturing settings, there is often a trade-off between maximizing yield and minimizing the per-realization cost, such as energy or time expenditure.^99^ To formalize this trade-off, we define a protocol cost function

2 $$C\left(\tau_{neq}\right)=\tilde{SA}\left(\tau^{neq}\right)-0.5{\mathrm{T̃}}_{Drive}\left(\tau^{neq}\right)$$

where $\tilde{SA}$ and ${\mathrm{T̃}}_{Drive}$ denote the normalized assembly yield and total drive activation time, respectively. Each is normalized by the maximum value observed across both MSM and KMC ensembles. The weighting factor of 0.5 reflects a practical assumption that yield is typically prioritized over cost, in line with trends in industrial applications.^100^ However, this coefficient is illustrative and may be adjusted based on specific objectives. The cost function *C*(τ_neq_) was evaluated using the yield and *T*_Drive_ metrics across various τ^neq^ values for both *N*_s_ values. As shown in Figure 7E,F, the MSM-predicted cost profiles closely follow the convex shape of the ground truth curves, with their minima appearing near the actual optima. This alignment highlights the ability of the driven MSM to correctly identify near-optimal control parameters.

Taken together, these results underscore the potential of the stochastic landscape-based MSM as an efficient surrogate model for control protocol optimization, providing reliable guidance based on outcome-based performance metrics.

### Computational Speedup and Broader Context of the SLM-MSM Approach

One of the primary advantages of the proposed MSM-based framework is its exceptional computational efficiency. For both equilibrium and driven scenarios with *N*_s_ = 26, the MSM simulations yielded results that closely mirrored those of full kinetic Monte Carlo simulations, while reducing computational time by approximately 10^7^ to 10^8^ fold. More concretely, ensemble-level statistics such as yield % SA, *T*_FAS_ distributions, and *T*_Drive_ were generated by the MSM within milliseconds. In contrast, the equivalent KMC simulations required several hours of computation to produce the same statistics across comparable ensemble sizes (see Section S5 of the Supporting Information for details).

This dramatic speedup underscores the power of the MSM framework as a surrogate model that enables fast and reliable exploration of parameter space, which is particularly valuable when optimizing control protocols. By dramatically reducing the computational burden without sacrificing predictive accuracy, the MSM approach opens the door to real-time, closed-loop optimization strategies for complex, nonequilibrium self-assembly systems.

The SLM–MSM rests on assumptions that can lead to limited performance in particular regimes. When distinct metastable basins exhibit nearly indistinguishable behavior in the chosen stochastic coordinate, e.g., similar mean energy and trend, they may be conflated into a single state, obscuring pathway differences on the route to the global minimum. Performance further depends on selecting a one-dimensional observable that tracks slow kinetics; when it is weakly informative, the method can fail. This limitation can be alleviated by constructing a more meaningful one-dimensional descriptor, as described before, and then re-estimating the MSM. Although Figure S3 shows correlations between other descriptors and the system’s energy, their potential utility for MSM construction remains to be established in future work. The SLM-MSM use of trap region to identify unvisited metastable states is heuristic, and thus extrapolation to unvisited basins should be validated against ground-truth or held-out trajectories for each system. Finally, in nonequilibrium control settings, poorly tuned resets in amplitude or frequency may destabilize rather than accelerate assembly. Practical mitigation of this failure mode involves a drive amplitude parameter sweep and choosing the most valuable value. It is also possible that our suggested drive scheme can generalize over drive amplitudes as well as durations, yet this is left for future work. Because the SLM–MSM pipeline is relatively lightweight, examining whether a system to be modeled yields these failure points can be performed quickly, compared to yield and first assembly times.

The SLM–MSM is intended to complement diffusion maps^101^ and deep-learning MSMs.^83–85^ Diffusion maps and deep-learning MSMs typically learn kinetics from large frame sets and perform best when all kinetically relevant basins are sampled. The SLM–MSM extracts a one-dimensional stochastic coordinate via trend segmentation and defines trap states by a heuristic rule, enabling MSM construction from modest trajectory collections. This heuristic can generalize trap identification to newly encountered basins with similar local statistics, which we have found useful on rugged landscapes, particularly under nonequilibrium driving conditions. The suggested approach has succeeded in generalizing beyond its learned data set to other nonequilibrium driving regimes, which is a unique merit of the SLM. The method also emphasizes interpretability, state boundaries are auditable and transition state theory motivated, and it has a small computational footprint that facilitates rapid iteration. We therefore view SLM–MSM as attractive when data are limited, nonequilibrium protocols are central, interpretability is required, and quick examination of the results is valuable.

## Conclusions

Coarse-graining complex stochastic systems remains a foundational challenge in statistical physics, particularly in systems governed by rare events and multiple interacting time scales. In this work, we introduce a data-driven yet physically grounded Markov state model (MSM) framework, built upon the stochastic landscape method (SLM), that addresses this challenge in a principled and computationally efficient manner. By combining insights from transition state theory with a modern change-point detection approach, the proposed MSM framework enables both predictive accuracy and mechanistic insight. We demonstrate that the resulting MSMs capture essential features of the underlying dynamics, including trend-dependent dwell times, imitation of the drive activation scheme, near-exponential waiting times, and robust prediction of observables. Notably, the MSM accurately reproduces key outcomes of both equilibrium and nonequilibrium self-assembly simulations, including the distribution of time to first assembly, yield, total drive activation duration, and a practical cost function across a range of drive protocols.

A notable strength of this framework is its reliance on a low-dimensional observable, specifically, a one-dimensional energy trajectory, rather than high-dimensional structural configurations. In contrast, many existing approaches rely on high-dimensional geometric descriptors or 2D structural snapshots, while our method is well-suited for relatively small, heterogeneous systems, where stochastic fluctuations and trend changes are prominent in the observable. The computational efficiency of the MSM is especially significant: simulation runtimes are reduced by 7–8 orders of magnitude compared to full kinetic Monte Carlo (KMC) simulations, without compromising accuracy. This speedup enables rapid exploration and optimization of self-assembly protocols that would otherwise be infeasible, supporting applications in real-time control and design of nonequilibrium processes.

Beyond predictive performance, the MSM also provides physical insight. By examining coarse-grained state transitions and dwell-time statistics, we identify meaningful mechanistic trends. In particular, dwell times scale strongly with the local trend variable, consistent with our earlier work,^11^ while drive-induced state switching is more sensitive to mean energy, with weaker dependence on trend. Furthermore, we demonstrate that a trend-triggered shock protocol can be effectively modeled using two equilibrium MSMs, representing the system before and after the drive, connected by level-jumping matrices that encode transitions between these regimes.

Given its accuracy, efficiency, and interpretability, the SLM-based MSM framework offers a promising foundation for studying and optimizing a wide range of complex, driven systems. Potential applications include viral capsid formation, multicomponent colloidal assembly, programmable materials, and scalable manufacturing protocols. Future extensions may incorporate a weighted SLM trajectory variable instead of the energy, non-Markovian dynamics, or leverage short-time simulations to extrapolate long-time behavior, following recent directions proposed in the literature.^40,42^ Future work may focus on developing an experimental system analogous to the one examined here, employing image-guided, laser-induced local heating to enable reversible assembly–disassembly cycles.^70^ Where kinetics permit, reversible photoacids could also be explored for feedback-timed bulk pH changes.^86^

In summary, our results establish the SLM-based MSM as a powerful baseline for surrogate modeling, analysis, and control of nonequilibrium self-assembly. It unifies data-driven simulation with physical insight, enabling scalable, interpretable exploration of stochastic dynamics in complex systems. More broadly, this framework offers a path toward simplifying and understanding complex, data-driven stochastic systems, not only for optimizing self-assembly but also for uncovering emergent behaviors in diverse physical, chemical, and biological contexts.

## Supplementary Material



## Data Availability

The data and software used to create this research are available upon request.

## Notes

A detailed description of the kinetic Monte Carlo (KMC) simulation method, including equilibrium modeling and rate constant estimation. It also outlines the parameter selection rationale, covering the minimal sampling criterion, KMC ensemble sizes, BEAST window time determination, default drive activation window selection, and the sizing of transition and level-jumping matrices. Additional supporting results and computational benchmarking are also provided. The supplementary figures and tables are listed below: Figure S1: System model overview, Figure S2: Segmented energy trajectories by the BEAST, Figure S3: Kinetic Monte Carlo (KMC) trajectories for six independent runs: Energies and geometrical descriptors, Figure S4: SA % vs τ^neq^ for the KMC simulation, Figure S5: Sensitivity of BEAST segmentation, Figure S6: Implied time scales and Chapman–Kolmogorov tests, Figure S7: *T*_dwell_ vs ⟨*t**⟩ for configuration 1, Figure S8: The MSM initial states heatmaps for configurations 1 and 2, Figure S9: Configuration 1 equilibrium MSM with *N*_s_ = 26 states, Figure S10: Configuration 2 equilibrium MSM with *N*_s_ = 26 states, Figure S11: The default MSM coarse-grained states snapshots, Figure S12: *T*_dwell_ histograms of configuration 1 equilibrium MSM, with *N*_s_ = 10 states, Figure S13: *T*_dwell_ histograms of configuration 2 equilibrium MSM, with *N*_s_ = 10 states, Figure S14: *T*_dwell_ histograms of configuration 1 equilibrium MSM, with *N*_s_ = 26 states, Figure S15: *T*_dwell_ histograms of configuration 2 equilibrium MSM, with *N*_s_ = 26 states, Figure S16: ⟨*T*_dwell_⟩ vs state index for configuration 2 equilibrium MSM with *N*_s_ = 26 states, Figure S17: Forward (*T*_F_) and backward (*T*_B_) transition matrices for the driven MSMs, Table S1: The default KMC simulation parameters, Table S2: The MSM simulation parameters, Table S3: Energy and trend bin assignments for the *N*_s_ = 10 MSMs, Table S4: Energy and trend bin assignments for the *N*_s_ = 26 MSMs (PDF)
