# Supplementary material for: Coarse-Graining Self-Assembly by the Stochastic Landscape Method
Source: J Chem Theory Comput. 2025 Oct 27;21(21):10719–34. doi: 10.1021/acs.jctc.5c01241 (PMC12613318; doi:10.1021/acs.jctc.5c01241)
Supplement: Supplementary file 1 [file ct5c01241_si_001.pdf]

Supporting Information

Coarse-Graining Self-Assembly by the Stochastic Landscape Method

Michael Faran[†] and Gili Bisker[*,†,‡,¶,§,‖]

†*School of Biomedical Engineering, Faculty of Engineering, Tel Aviv University, Tel Aviv 69978, Israel*

‡*The Center for Physics and Chemistry of Living Systems, Tel Aviv University, Tel Aviv 6997801, Israel*

¶*The Center for Nanoscience and Nanotechnology, Tel Aviv University, Tel Aviv 6997801, Israel*

§*The Center for Light-Matter Interaction, Tel Aviv University, Tel Aviv 6997801, Israel*

‖*The Center for Computational Molecular and Materials Science, Tel Aviv University, Tel Aviv 6997801, Israel*

E-mail: bisker@tauex.tau.ac.il

S1 The Kinetic Monte Carlo Simulation

S1.1 Equilibrium Self-Assembly Model

We simulate nonequilibrium self-assembly using the kinetic Monte Carlo (KMC) method,[1] which is mathematically equivalent to the Gillespie algorithm.[2] This enables us to incorporate realistic time scales into the dynamics, providing an advantage over earlier discrete-time models used in prior work.[3,4]

Our framework explores the self-assembly behavior of N uniquely labeled particles ($a = 1, \ldots, N$), each capable of switching between internal states $s_a = 1, \ldots, M_{\mathrm{T}}$, where each state corresponds to a distinct target configuration. This approach is inspired by biomolecular systems, in which components undergo conformational changes as they assemble into complex structures. As depicted in Figure S1A, the simulation begins with particles placed at random positions on an $L \times L$ lattice, ensuring that each lattice site is either unoccupied or occupied by a single particle with a random initial state. The desired target configurations, illustrated in Figure S1B, are specific spatial arrangements involving all particles, defined through their pairwise interaction energies. These configurations correspond to the system's global energy minima and represent the intended outcomes of the self-assembly process.

Particles interact via pairwise potentials $J(s_a, s_b)$ that depend on their internal states and the structure of the encoded targets. These interactions are strongest when a particle pair adopts a state combination that aligns with a target configuration, and weaker when the pairing deviates from the target or involves unrelated neighbors. Two particles are considered nearest neighbors if they occupy adjacent sites on the lattice, i.e., if they share a common edge. A given particle pair is labeled as "neighboring" if they appear as adjacent in at least one of the predefined target structures (see Figure S1B). The magnitude of the interaction between two particles is determined based on the following criteria:

- Strong attraction (J_s): When two adjacent particles on the lattice share the same internal state corresponding to a target structure ($s_a = s_b = m$) and are marked as

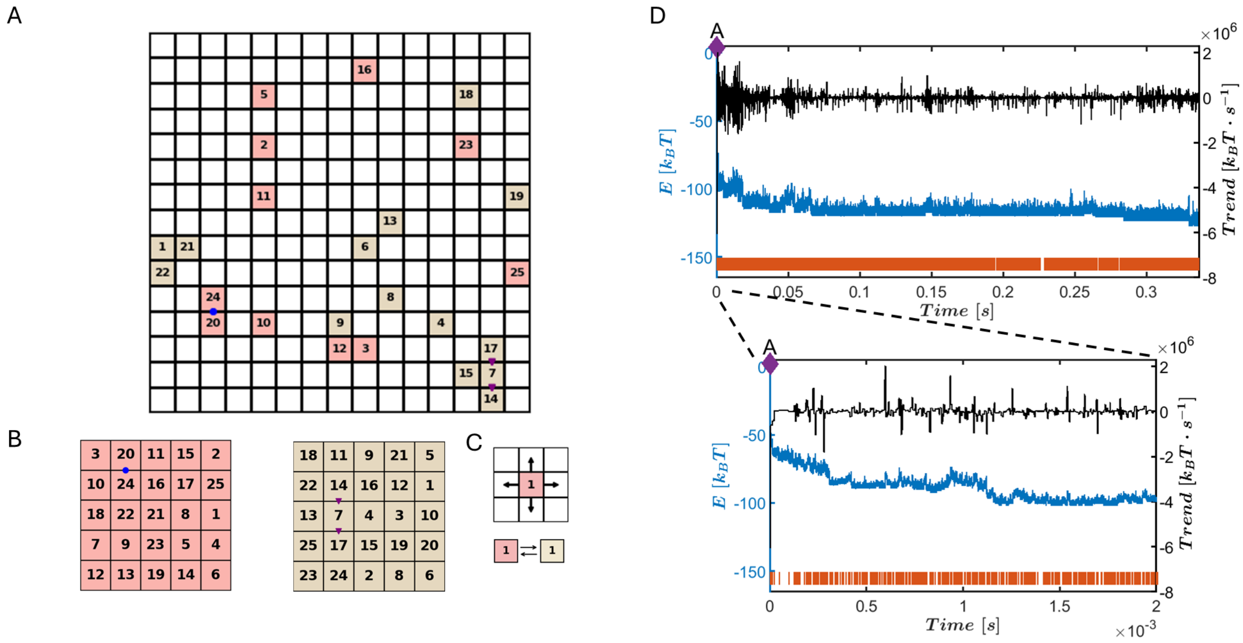

Figure S1: System model overview. Panels are arranged top-to-bottom, then left-to-right. (A) A 15×15 grid ($L = 15$) contains $N = 25$ uniquely labeled particles, each occupying a site and adopting one of $M_{\mathrm{T}} = 2$ possible internal configurations (colored red or brown). Purple triangles and blue circles between adjacent particles represent specific bonding interactions, reflecting a predefined target pair that exhibits a strong interaction. The visualized configuration shows the system's initial state, chosen randomly. (B) Two distinct target assemblies with minimal energy, each specifying a different spatial arrangement of the N particles. (C) During each simulation step, the system randomly selects a move from a predefined set, either a spatial relocation on the grid or a switch between internal states. The probabilities for each move are updated dynamically in response to the system's current configuration. (D) An example of the system's energy trajectory for configuration 1 ($J_s = -3.6$ [k_BT] and $J_w = -1$ [k_BT]), along with corresponding trend values shown in black (referencing the right axis). The energy trajectory is segmented in real-time using the BEAST algorithm; each segment is bounded by orange vertical lines and assigned a mean trend value, $\langle t^* \rangle$. The top plot displays the whole trajectory, while the lower panel offers a magnified view of a selected interval. The purple diamond labeled 'A' marks the simulation time at which the configuration in panel (A) occurs.

neighbors in that target m, they interact with a strong attractive force. This also applies if they have different internal states ($s_a = m, s_b = n, m \neq n$) but are adjacent in both target structures m and n.

- Weak attraction (J_w): A weak interaction occurs if the particle pair is not adjacent in any target configuration, or if their current states do not correspond to any shared target where they are considered neighbors.
- Intermediate attraction ($\frac{J_s+J_w}{2}$): If the particles are neighbors in one target configuration m (but not in another $n \neq m$), and their states are ($s_a = m, s_b = n$), they experience an interaction of intermediate strength.

Thus, the i and j particle interaction can be rewritten as:

$$J(s_a, s_b) = J_w + \sum_{m=1}^{M_{\mathrm{T}}} I_{a,b}^{m} (\frac{J_s - J_w}{2})(\delta_{m,s_a} + \delta_{m,s_b}), \tag{S.1}$$

where $I_{a,b}^{m}$ denotes the adjacency matrix for target structure m, such that $I_{a,b}^{m} = 1$ if particles a and b are defined as neighbors in target m, and 0 otherwise. The Kronecker delta δ_{m,s_a} equals 1 when the internal state of particle a matches m, and 0 otherwise. We also define $B(a, b)$ as the dynamic adjacency matrix of the lattice, where $B(a, b) = 1$ indicates that particles a and b are currently nearest neighbors on the lattice, and 0 otherwise. With this definition, the total interaction energy of the system, E, is then given by:

$$E = \frac{1}{2} \sum_{a \neq b}^{N} \sum_{b=1}^{N} J(s_a, s_b) B(a, b). \tag{S.2}$$

Following Eqs. (S.1) and (S.2), strong bonds, i.e., edges that attain J_s, occur when neighbor particle pairs (a, b) attain $B(a, b) = 1$, $I_{a,b}^{m} = 1$ for some m, and $s_a = s_b = m$.

At each step of the simulation, a particle may either relocate to an adjacent lattice site or switch its internal state, simulating a conformational shift (see Figure S1C). The set of possible moves and their corresponding rates are recalculated dynamically based on the

system's current configuration. We employ the n-fold way variant of the kinetic Monte Carlo algorithm[1] to stochastically determine the next event. This method enables the system to evolve naturally along its energy landscape, capturing the continuous and physically realistic dynamics of the assembly process.

The simulation is initialized from a randomly chosen configuration, specifying both the spatial positions and internal states of the N particles (see example in Figure S1A). At each step of the kinetic Monte Carlo (KMC) process, the system considers a total of $N_{\text{tot}} = 4N + (M_{\text{T}} - 1)N = (3 + M_{\text{T}})N$ possible transitions. These include $4N$ possible single-particle movements (one for each direction in 2D space) and $(M_{\text{T}} - 1)N$ internal state transitions, where each particle may switch to any of the other $M_{\text{T}} - 1$ states.

For translational moves, the transition rate r_{cd} from a configuration d (with energy E_d) to a configuration c (with energy E_c) is computed using an energy-sensitive formulation, as commonly used in lattice-based dynamics:[5–9]

$$r_{cd} = r_0 \exp\left(-\frac{E_c - E_d}{2k_B T}\right). \tag{S.3}$$

where, k_B denotes the Boltzmann constant, T is the temperature, and all energy values are expressed in units normalized by these parameters. Translations that would result in a particle attempting to move into an already occupied site are always rejected, and assigned a transition rate of $r_{cd} = 0$.

We define a base rate constant $r_0 \approx 2.5 \times 10^6\ [s]^{-1}$, which serves as a reference for scaling all movement rates. This value was estimated based on the diffusion time required for a particle to traverse a distance equivalent to its diameter, analogous to moving between adjacent lattice tiles. This time scale reflects the free diffusion limit, where there is no net energy change between the initial and final states ($E_c = E_d$). Further details on the derivation of r_0 can be found in Subsection S1.2.

Analogously, for internal state transitions (relevant when $M_{\text{T}} > 1$), the rates q_{cd} are given

by:

$$q_{cd} = \frac{4r_0}{M_\mathrm{T} - 1} \exp\left(-\frac{E_c - E_d}{2k_BT}\right). \tag{S.4}$$

It is important to note that in our simulations, translational and internal state-switch moves are designed to have comparable probabilities for a given particle when the energy remains unchanged ($E_c = E_d$). The prefactor $4r_0$ accounts for the four directional movement options in two dimensions, while the denominator $M_\mathrm{T} - 1$ corresponds to the number of alternative internal states available for switching.

The cumulative rate function R_{cd} used in the KMC algorithm to select the next event and propagate the system is given by:

$$R_{cd} = R_{4N,j}\Theta(c - 4N) + \sum_{m=1}^{c} r_{md}\Theta(4N - c) + \sum_{m=4N}^{i} q_{md}, \tag{S.5}$$

where Θ denotes the Heaviside step function. The cumulative rate function is constructed so that for indices $i > 4N$, only internal state-switch moves are included in the KMC selection process. In contrast, indices $i \leq 4N$ correspond exclusively to translation moves.

The total escape rate from the current state d is given by $Q = R_{N_\mathrm{tot},d}$. To determine which move occurs, a uniform random number $u \in (0, 1]$ is drawn, and the selected move index $i = I$ satisfies the condition $R_{I-1,d} < uQ \leq R_{I,d}$. After the transition, a second random number $u' \in (0, 1]$ is used to compute the time increment as $\Delta t_h = Q^{-1} \ln\left(\frac{1}{u'}\right)$, where h indexes the KMC steps, starting from $h = 1$ up to a final count of $h = N_\mathrm{steps}$. Accordingly, the total simulation time is calculated as $T_\mathrm{tot} = \sum_{h=1}^{N_\mathrm{steps}} \Delta t_h$. Unless stated otherwise, the KMC simulation default parameters follow Table S1.

As described in the main text, following the blue arrows in Figure 2A, the resulting energy trajectory of the KMC simulation is divided in real-time into sub-trajectories with duration $\tau_{\mathrm{BEAST},i}$, where i is the configuration number as in the main text. The rationale for choosing the value of $\tau_{\mathrm{BEAST},i}$ is detailed in Subsection S2.3.

These sub-trajectories are segmented using the Bayesian Estimator of Abrupt Changes,

Table S1: The Default Simulation Parameters

Parameter	Notation	Value
Number of Particles	N	25
Grid Size	L	15
Number of Targets	M_{T}	2
Strong Energy	J_s	-3.6 $[k_B T]$
Weak Energy	J_w	-1 $[k_B T]$
Simulation Time Cap	N_{steps}	5×10^7

Seasonality, and Trend (BEAST) algorithm,[10] as implemented in our previous studies.[4,11,12] For each resulting segment, the mean energy, $\langle E^* \rangle$, and mean trend $\langle t^* \rangle$, are computed. The mean trend is computed by averaging the differences between each two consecutive energy values in the segment.

A representative example of an equilibrium energy trajectory with its segmented intervals and corresponding mean trends over time is shown in Figure S1D, including a zoomed-in view for clarity. Additional zoomed-in examples are depicted in Figure S2. The energy segmentation and stochastic coordinate extraction are performed independently of the physical simulation, and they do not interfere with the equilibrium simulation process.

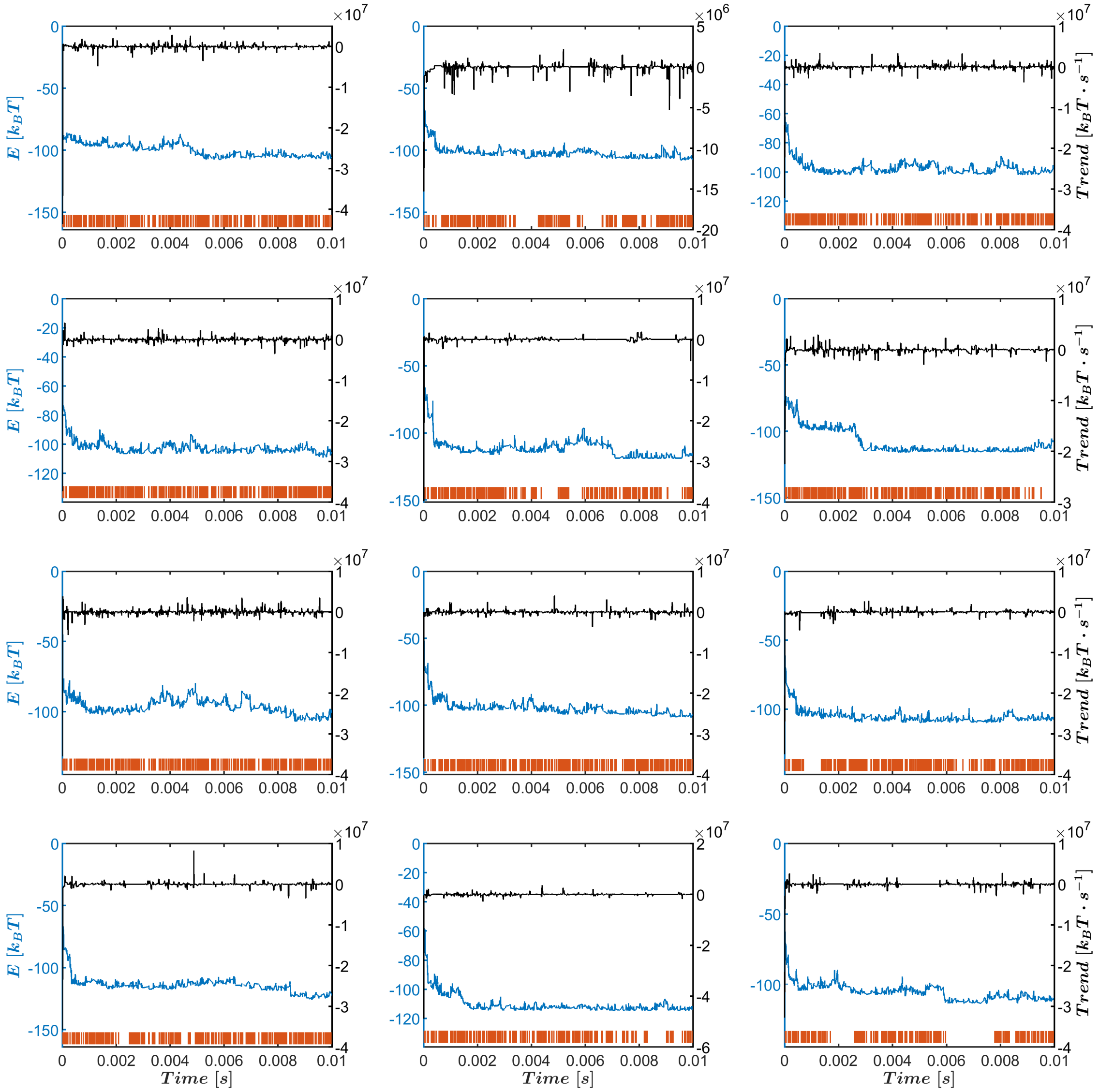

Figure S2: Segmented energy trajectories by the BEAST. Energy-time trajectories of 12 simulation runs are shown for the default parameters of Table S1, all zoomed up to 0.01 [s]. The raw energy traces are plotted in blue, with their temporal trends overlaid in black. Orange vertical lines indicate the segment boundaries detected by the BEAST algorithm, as detailed in the main text.

S1.2 Rate Constant Calculation

The characteristic time scale for a protein to diffuse a distance comparable to its own size in a cellular environment can be estimated using the mean-squared displacement relation for Brownian motion. For a typical globular protein with a radius of gyration around 2 $[nm]$,[13] the diffusion coefficient within the cytoplasm is approximately 10 $[\mu m]^2/[s]$, roughly an order of magnitude slower than in pure water.[14] Applying the standard diffusion relation $\tau \approx x^2/D$,[15] where x is the diffusion distance and D the diffusion coefficient, we estimate the time required for a protein to travel its diameter (approximately 4 $[nm]$) as: $\tau \approx (4\ [nm])^2/(10\ [\mu m]^2/[s]) \approx 4 \times 10^{-7}\ [s]$ or 0.4 $[\mu s]$.

The corresponding rate constant r_0, defined as the inverse of this time scale, is $r_0 \approx 1/\tau \approx 2.5 \times 10^6\ [s]^{-1}$. This value serves as a biologically grounded reference time scale for diffusive events such as molecular or protein-protein interactions, and conformational changes within the cell.[16] It provides the foundation for calibrating movement and state-transition rates in our KMC simulations of self-assembly dynamics.

S1.3 Energy and geometric descriptors

Although in this work the energy trajectory was chosen as the baseline KMC observable for SLM post–processing, other choices are possible, as discussed in the main text. To demonstrate that the total energy is a suitable descriptor for this system, we compare its trajectory with commonly used geometric measures for metastability in self-assembly.[17] Specifically, we consider (i) the dispersion degree (DD),

$$\mathrm{DD}(t) \;=\; 1 - \frac{n_{\max}(t)}{N}, \qquad \text{(S.6)}$$

where $n_{\max}(t)$ is the size of the largest cluster at time t among N particles, and (ii) a 2D surrogate of the solvent–accessible surface area (SASA) that we term the normalized

exposed tile edges (NETE). Let ETE(t) be the number of exposed edges at time t (i.e., edges adjacent to empty lattice sites). For a system of N tiles on a square lattice, the maximally exposed state has max(ETE) $= 4N$, while the assembled square target (for $N = 25$) has min(ETE) $= 20$. We therefore define

$$\mathrm{NETE}(t) \;=\; \frac{\mathrm{ETE}(t) - \min(\mathrm{ETE})}{\max(\mathrm{ETE}) - \min(\mathrm{ETE})} \;\in\; [0,1]. \tag{S.7}$$

For a consistent comparison, we also normalize the energy,

$$\hat{E}(t) \;=\; \frac{E(t) - \min(E)}{\max(E) - \min(E)}, \tag{S.8}$$

with $\max(E) = 0$, $\min(E) = -140$ $[k_BT]$. Analogously, the NETE shown in the figures corresponds to an affine mapping of the raw series to $[0, 1]$.

We visualize the six trajectories obtained with the default KMC parameters in Figure S3 (downsampled by a factor of 100 for clarity). For each run, we report the Pearson correlation coefficients between the normalized energy and the two geometric descriptors, $r_{\hat{E},\mathrm{NETE}}$ and $r_{\hat{E},\mathrm{DD}}$, computed on the downsampled series. Averaged over the six examples, we obtain $\overline{r}_{\hat{E},\mathrm{NETE}} = 0.82$ and $\overline{r}_{\hat{E},\mathrm{DD}} = 0.76$, indicating strong correlations between energy and structural measures. The higher correlation with NETE is expected because its minimum coincides one-to-one with the minimum-energy assembled target, whereas DD $= 0$ can also occur in other configurations. Overall, these results support the validity of the energy as a one-dimensional descriptor for this system, alongside geometric descriptors such as NETE and DD.

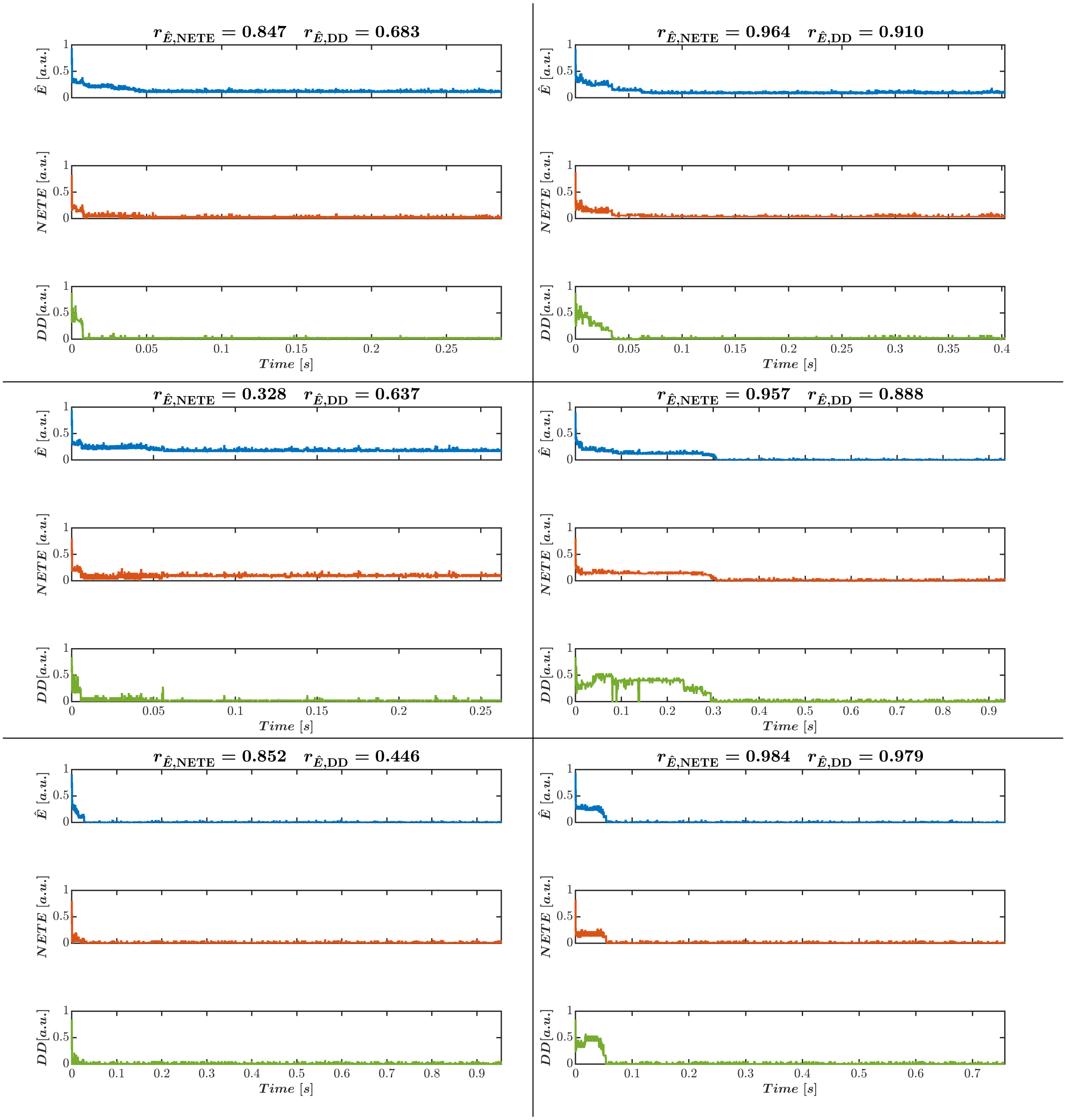

Figure S3: Kinetic Monte Carlo (KMC) trajectories for six independent runs of the lattice self-assembly model using the default simulation parameters. Each macro-panel (three vertically stacked subpanels) shows the time evolution of: (top) the normalized energy $\hat{E}$ (blue), (middle) the normalized exposed tile edges (NETE, orange), and (bottom) the dispersion degree (DD, green), as defined in the text. All observables are scaled to the interval $[0, 1]$ and plotted against the continuous simulation time (seconds), enabling direct visual comparison within each macro-panel. Above each macro-panel we report Pearson correlations between $\hat{E}$ and the two structural descriptors, $r_{\hat{E},\mathrm{NETE}}$ and $r_{\hat{E},\mathrm{DD}}$, computed from the downsampled trajectories.

S2 Parameter Selection Rationale

This section outlines the rationale behind the selection of key simulation parameters used throughout this study. A summary of the main parameter definitions and their corresponding values, as applied in the construction of the Markov state models (MSMs), is provided in Table S2. This overview serves as a reference for interpreting the simulation setup and its connection to the MSM-based coarse-graining framework.

Table S2: The MSM Simulation Parameters

Parameter	Notation	Value
Downsample Index	d_s	100
Configuration Number	i	1 or 2
BEAST Activation Time scale	$\tau_{\text{BEAST},i}$	3.7 $[ms]$ for $i=1$ and 0.54 $[ms]$ for $i=2$
Drive Activation Time Used for Learning	$\tau_{\text{L}}^{\text{neq}}$	$0.12, 0.25, 0.5, 1, 1.5, 2, 2.5, 2.9$ $[ms]$
Number of MSM States	N_s	10 or 26
Stochastic Coordinates Bin Number	n_{bins}	3 or 5
Drive Amplitude	ρ	1.5
Tested Drive Activation Time	τ^{neq}	0.12 $[ms]$
Equilibrium KMC Trajectory Number	N_i^{eq}	300 for $i=1$ and 60 for $i=2$
Equilibrium Segments Set	Ω_i^{eq}	Determined by configuration i results
Nonequilibrium KMC Trajectory Number	N^{neq}	420
Nonequilibrium Segments Set	Ω^{neq}	Determined by results
Segment Mean Energy	$\langle E^* \rangle$	Determined by results per segment
Segment Mean Trend	$\langle t^* \rangle$	Determined by results per segment
Segment Dwell Time	T_{dwell}	Determined by results per segment
The Trap Region	T^*	Determined by results
Forward Level-jumping Matrix	T_{F}	Determined by results
Backward Level-jumping Matrix	T_{B}	Determined by results
Total Drive Activation Time	T_{Drive}	Determined by results
Minimal Samples Number	n_{bound}	Determined by results

We introduce a statistically grounded estimation criterion to ensure the reliability of key parameters. This minimal sampling rule establishes a clear threshold for statistical validity by specifying the minimum number of observations required to achieve an acceptable level of estimation error. This criterion serves as a foundation for selecting appropriate ensemble sizes and transition statistics throughout the modeling process. Based on this rule, we first justify the choices of N_1^{eq} and N_2^{eq}, the number of equilibrium simulations used for

configurations 1 and 2. We then present the rationale behind the selection of characteristic time scales, including the BEAST activation window $\tau_{\mathrm{BEAST},i}$ for configurations $i = 1, 2$, and the drive duration $\tau_{\mathrm{L}}^{\mathrm{neq}}$ used in the driven simulations. Finally, we apply the minimal sampling criterion to the evaluation of the MSM transition matrices for both configurations, as well as to the forward-level jumping matrix T_{F}, the number of driven simulations N^{neq}, and the backward-level transition matrix T_{B}. These analyses collectively ensure the statistical robustness of the MSM construction across equilibrium and nonequilibrium regimes.

S2.1 Minimal Sampling Criterion

Assume a random vector $\vec{p}$ representing a discrete probability distribution (histogram). To estimate this distribution from data correctly, we require that each empirical frequency of the vector $\hat{\vec{p}}$ approximates the actual underlying bin of the distribution p with bounded error. Specifically, we wish to ensure that the maximum per-entry error in the vector satisfies $\max_g |\hat{p}_g - p_g| \leq \epsilon$ with high probability. Let n_{bound} be the minimal sample size required for estimation of $\vec{p}$ within the minimal sampling criterion.

To achieve this, we apply Hoeffding's inequality[18] to bound the deviation in each bin, followed by a union bound over all $|\vec{p}|$ entries. For n_{bound} independent samples from the distribution, Hoeffding's inequality[18] gives:

$$\Pr\left(|\hat{p}_g - p_g| \geq \epsilon\right) \leq 2\exp(-2n_{\mathrm{bound}}\epsilon^2). \tag{S.9}$$

Applying a union bound over all $|p|$ entries gives:

$$\Pr\left(\exists g\ \text{, such that}\ |\hat{p}_g - p_g| \geq \epsilon\right) \leq 2|\vec{p}|\exp(-2n_{\mathrm{bound}}\epsilon^2). \tag{S.10}$$

To guarantee that the total probability of deviation does not exceed δ, we impose the condition: $2|\vec{p}|\exp(-2n_{\mathrm{bound}}\epsilon^2) \leq \delta$. Rearranging yields the required number of samples:

$$n_{\text{bound}} \geq \frac{1}{2\epsilon^2} \log\left(\frac{2|\vec{p}|}{\delta}\right). \tag{S.11}$$

where, in our case, $\epsilon = 0.2$, $\delta = 0.2$ are chosen for the criterion. Thus, depending on the size of $|\vec{p}|$, n_{bound} is found using equation (S.11).

S2.2 KMC Simulation Ensembles Sizes

To ensure statistically reliable histograms for the resulting T_{FAS} distributions, we required a minimum of $n_{\text{bound}} = 59$ successful assembly events. This threshold was derived from the minimal sampling criterion in Eq. (S.11), assuming a maximum of 10 histogram bins. We initially simulated $N_1^{\text{eq}} = 50$ and $N_2^{\text{eq}} = 50$ trajectories for configurations 1 and 2, respectively, using the equilibrium KMC scheme shown in Figure 2A of the main text. These simulations yielded assembly success rates of approximately 20% for configuration 1 and 100% for configuration 2. To meet the sampling requirement of $n_{\text{bound}} < N_i^{\text{eq}} \cdot (\%SA/100)$, we increased the ensemble sizes accordingly: N_1^{eq} was set to 300, and N_2^{eq} to 60. These choices guaranteed at least 59 successful assemblies in both cases, satisfying the minimal sampling criterion. Consequently, the histograms shown in Figure 5 in the main text, each constructed with fewer than 10 bins, are well-sampled and statistically representative of the underlying T_{FAS} distributions.

S2.3 Determination of the BEAST Window Duration

The value of $\tau_{\text{BEAST},i}$, which sets the temporal resolution for drive activation analysis in configuration i, is determined from a preliminary equilibrium KMC ensemble. For the default setup (configuration $i = 1$), the simulation parameters are provided in Table S1. For configuration $i = 2$, $\tau_{\text{BEAST},i}$ is recalibrated to reflect the modified interaction strengths of $J_s = -2.4$ $[k_BT]$ and $J_w = -0.67$ $[k_BT]$. To compute $\tau_{\text{BEAST},i}$, we collect the time-to-first-assembly (T_{FAS}) values from all trajectories in a preliminary equilibrium KMC ensemble.

For trajectories that do not reach an assembled state within the simulation window, we take the final simulation time as T_{FAS}. Following the procedure established in our prior work,[12] $\tau_{\mathrm{BEAST},i}$ is defined as 1% of the median T_{FAS} across the ensemble, resulting in $\tau_{\mathrm{BEAST},1} = 3.7$ milliseconds for configuration $i = 1$, and $\tau_{\mathrm{BEAST},2} = 0.54$ milliseconds for configuration $i = 2$. This selection ensures that the BEAST activation window captures relevant fluctuations in system dynamics, while remaining consistent across configurations with differing assembly kinetics.

S2.4 Selection of the Default Drive Activation Window

In the driven scheme, the drive activation window $\tau_{\mathrm{L}}^{\mathrm{neq}}$ used for training and constructing the driven MSM is selected from among the τ^{neq} values analyzed in the ground truth comparisons presented in the Results and Discussion section of the main text. The chosen value, $\tau_{\mathrm{L}}^{\mathrm{neq}} = 0.034 \cdot \tau_{\mathrm{BEAST},1}$, was determined heuristically to ensure effective construction of the driven MSM using the KMC-generated ensemble as a training baseline. To accurately model the driven assembly process, the MSM relies on statistically representative sampling of transitions into and out of each coarse-grained state across both levels, namely, the equilibrium MSMs of configurations 1 and 2. These transitions are captured by the level-jumping matrices T_{F} and T_{B}, which encode the system's dynamics during drive activation and deactivation. However, sampling these transitions poses a tradeoff. If $\tau_{\mathrm{L}}^{\mathrm{neq}}$ is too short, leading to rapid success and 100% yield, trajectories terminate quickly after only a few drive activations. This limits the ability to sample sufficient level-jumping transitions. On the other hand, if $\tau_{\mathrm{L}}^{\mathrm{neq}}$ is too long, producing near-zero yield, the system may never approach the assembled state, and transitions between near-target coarse-grained states remain unvisited. To balance these competing constraints, we selected the $\tau_{\mathrm{L}}^{\mathrm{neq}}$ value that produced a yield closest to 50% for the case of $N_s = 26$ states in each MSM level (see Figure S4). This intermediate yield ensures both long trajectories with frequent drive activations and sufficient sampling of transitions near the target state. This choice enables the statistically robust construction

of the level-jumping matrices, supporting accurate replication of the driven KMC scheme by the MSM framework.

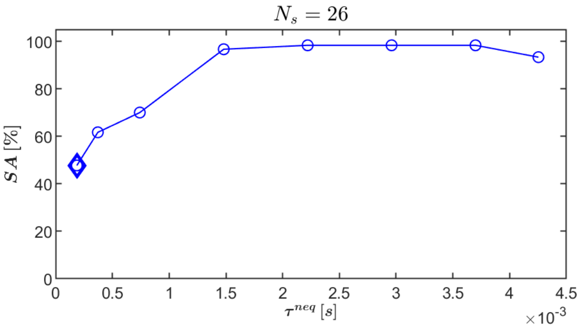

Figure S4: Driven assembly yield ($\%SA$) as a function of the drive activation window duration (τ^{neq}) in KMC simulations. Circular markers indicate the simulated values of τ^{neq} and their corresponding assembly yields. The diamond marker highlights the selected training value, $\tau_{\mathrm{L}}^{\mathrm{neq}}$, chosen to produce a yield closest to 50%, ensuring balanced sampling for constructing the driven MSM.

S2.5 Transition and Level-jumping Matrices Sizes

To construct the transition matrices for the equilibrium MSMs of configurations 1 and 2 with statistically reliable estimates, we treat each row as a discrete probability distribution vector $\vec{p}$ and apply the minimal sampling condition defined by Eq. (S.11). For the chosen MSM resolution of $N_{\mathrm{s}} = 26$ states, the number of possible transitions from each state is $|\vec{p}| = 26$. Substituting this value into Eq. (S.11) yields a minimal sample size requirement of $n_{\mathrm{bound}} = 69$ transitions per row to ensure statistical accuracy. Accordingly, the transition probability matrices for both equilibrium MSMs must be constructed using ensembles large enough to guarantee at least 69 observed transitions from each state. Using the selected KMC ensemble sizes N_1^{eq} and N_2^{eq} (see the equilibrium scheme in Figure 2A of the main text and corresponding segmentation procedure in Figure 2B of the main text), we confirm that this sampling criterion is met across all states, thus ensuring the reliability of the equilibrium transition matrices.

For the driven MSM, a similar minimal sampling requirement is imposed on the level-

jumping forward matrix T_{F}. Here, each row corresponds to a coarse-grained state in configuration 1 and encodes the probability of transitioning to a state in configuration 2 upon drive activation. As with the equilibrium matrices, we require $n_{\mathrm{bound}} = 69$ samples per row for $N_{\mathrm{s}} = 26$ to achieve statistical validity. However, empirical inspection reveals that certain coarse-grained states, particularly those with low mean energy and near-zero trend near the target configuration (e.g., state 11 in Table S4), are visited infrequently in the driven simulations. Therefore, achieving sufficient sampling across all rows necessitates a larger ensemble size. We find that, for the chosen value of $\tau_{\mathrm{L}}^{\mathrm{neq}}$, a driven simulation ensemble of $N^{\mathrm{neq}} = 420$ satisfies the minimal sampling condition for all rows of T_{F}.

In contrast, the backward level-jumping matrix T_{B}, estimated from Ω^{neq}, poses a greater challenge. Since T_{B} captures transitions from configuration 2 back to configuration 1 after drive deactivation, some states, especially those far from the target, are only rarely visited. As a result, enforcing the $n_{\mathrm{bound}} = 69$ criterion uniformly across T_{B} would require prohibitively large ensembles. Instead, we accept that T_{B} does not meet the minimal sampling condition and assess its accuracy qualitatively. As shown in Figures S17B and S17D, rows of T_{B} corresponding to different source states with similar mean energies exhibit consistent transition patterns, suggesting a degree of robustness despite sparse sampling. This observation is supported by the similar "after" state distributions seen in the colormaps and the associated state energy values listed in Tables S3 and S4. Importantly, even though T_{B} is the only matrix in the driven MSM that does not strictly satisfy the sampling criterion, the predictive performance of the model remains strong. As demonstrated in Figure 6 and Figure 7 of the main text, the driven MSM successfully reproduces key features of the ground truth dynamics, validating the overall framework.

S2.6 Sensitivity of BEAST to Window Size, Downsampling, and Noise

To assess the robustness of the BEAST segmentation used to extract the stochastic coordinates, we performed a one–at–a–time sensitivity analysis with respect to the activation window $\tau_{\mathrm{BEAST},1}$, the uniform downsampling index d_s, and additive white Gaussian noise (AWGN) on the observable. Aside from these three knobs, BEAST was run with a linear-trend, no-seasonality specification; the maximum number of admissible changepoints was set equal to the signal length (i.e., effectively unbounded), and the reported segmentation used the median of the posterior number of trend changepoints returned by BEAST. All remaining BEAST options were left at their default settings[10] and require no additional user tuning.

As a representative input we used the energy vs. time trajectory from Figure S3 (sixth panel), chosen because it contains a clear target assembly event under the default simulation configuration. Default KMC parameters are listed in Table S1, along with their rationale for choice.

The baseline configuration uses $\tau_{\mathrm{BEAST},1} = 3.7$ ms and $d_s = 100$ (see Table S2). The segmentation yields, for each detected segment, the mean energy $\langle E^* \rangle$ and the mean trend $\langle t^* \rangle$. We compare the empirical distributions of these coordinates across sensitivities by the histogram intersection (HI),[19]

$$\mathrm{HI}(p', q') = \sum_b \min\bigl(p_b,\, q_b\bigr), \tag{S.12}$$

where p_b and q_b are bin–probabilities in bin b of histograms p' and q'. To avoid bin–choice bias, bin widths are set once from the baseline data via Scott's rule,

$$h = 3.5\,\hat{\sigma}_{\mathrm{samples}}\, n_{\mathrm{samples}}^{-1/3}, \tag{S.13}$$

with $\hat{\sigma}_{\text{samples}}$ and n_{samples}, the baseline sample standard deviation and size, and the same bin edges are used for all tested settings (edges are expanded to span the global min/max across settings).

For the noise study we inject AWGN with total noise energy $E_n = \alpha E_s$ (relative to the signal energy $E_s = \sum_t E(t)^2$), giving $\text{SNR} = 10\log_{10}(E_s/E_n) = 10\log_{10}(1/\alpha)$. In a one–factor–at–a–time design, we first varied the BEAST window by halving and doubling the baseline $\tau_{\text{BEAST},1}$ while keeping $d_s = 100$. Next, we changed only the sampling resolution to $d_s \in \{50, 200\}$ at the baseline $\tau_{\text{BEAST},1}$. Finally, with $\tau_{\text{BEAST},1}$ and $d_s = 100$ fixed, we injected additive white Gaussian noise (AWGN) with energy ratios $\alpha \in \{0.01, 10^{-5/10}\}$, corresponding to $\text{SNR} \in \{20\,\text{dB}, 5\,\text{dB}\}$. For each case we report HI relative to the baseline histogram.

Figure S5 summarizes the results. Relative to baseline (HI = 1), halving/doubling $\tau_{\text{BEAST},1}$ causes modest changes in the distributions (trend: HI ≈ 0.82 ,0.88; energy: HI $\approx 0.93, 0.94$). Changing only the resolution to $d_s = 50$ or $d_s = 200$ also preserves good overlap (trend: HI $\approx 0.92, 0.93$; energy: HI $\approx 0.94, 0.95$). Noise is the dominant factor: at 20 dB SNR we still observe moderate overlap (trend HI$\approx$ 0.78, energy HI $\approx$ 0.81); at 5 dB SNR the energy distribution broadens noticeably (trend HI $\approx$ 0.76, energy HI$\approx$ 0.58). These outcomes are consistent with the visual impression of the histograms similarity with respect to the baseline.

Overall, within reasonable ranges, the BEAST outputs used by the SLM are stable to τ_{BEAST} and d_s, supporting the defaults $\tau_{\text{BEAST},1} = 3.7$ [ms] and $d_s = 100$. While high noise levels (5 dB SNR) degrade the overlap, especially for $\langle E^* \rangle$, the segmentation remains qualitatively faithful at 20 dB SNR. These tests justify the parameter choices employed throughout the work and quantify robustness under noise.

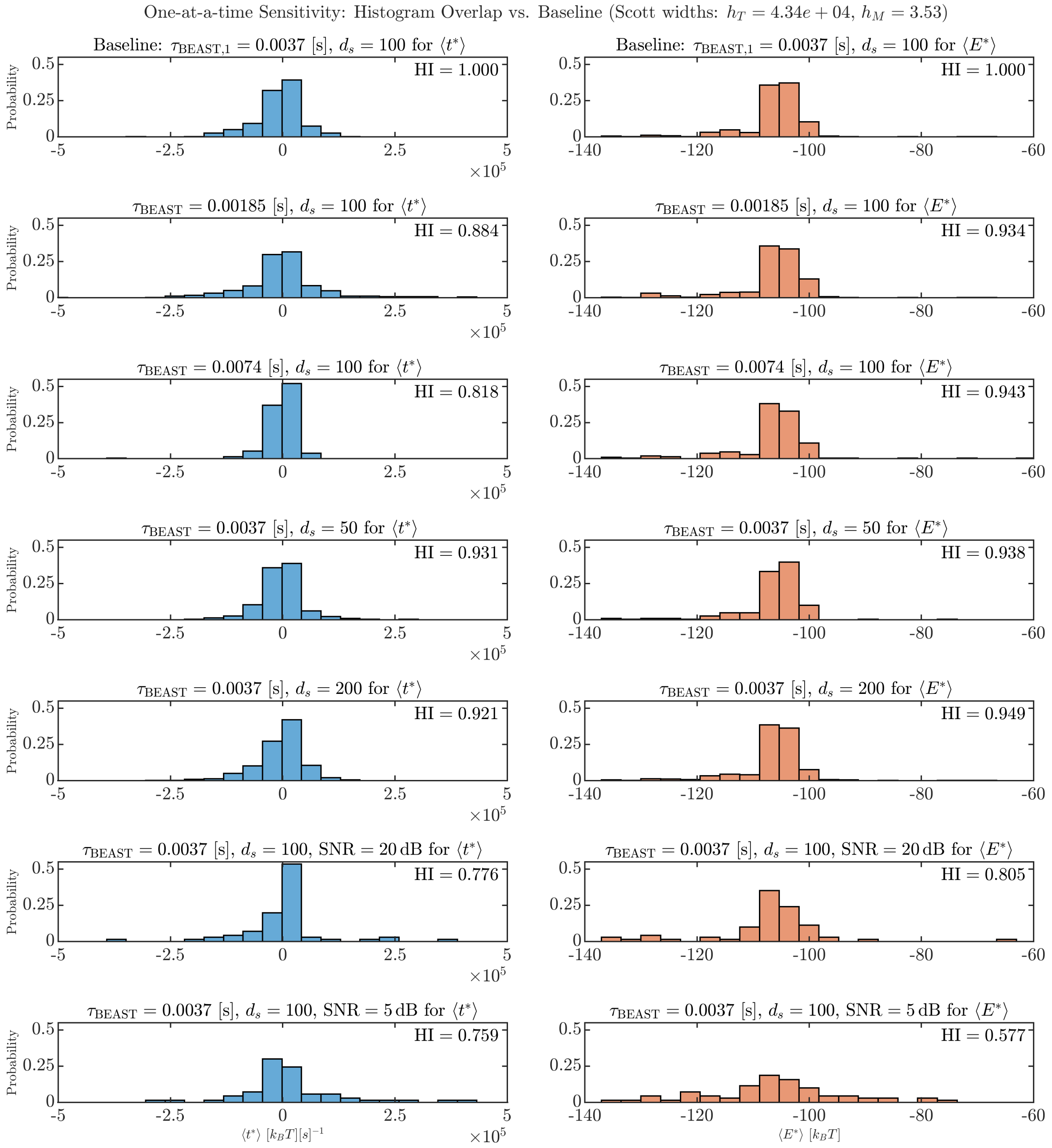

Figure S5: **Sensitivity of BEAST segmentation.** One–at–a–time changes to $\tau_{\mathrm{BEAST},1}$, d_s, and AWGN level (reported as SNR in dB) applied to the energy vs. time trajectory from Figure S3 (sixth panel: third row, column two). Left column: probability histograms of $\langle t^* \rangle$. Right column: histograms of $\langle E^* \rangle$. All histograms use Scott's bin width computed from the baseline and shared bin edges; the overlap score in each panel is the histogram intersection HI with the baseline. Defaults are $\tau_{\mathrm{BEAST},1} = 3.7$ ms and $d_s = 100$.

S3 The Markov State Model Simulation - Additional Details

S3.1 Percentile-Based Coarse-Graining

To construct the Markov state model (MSM), we first define a coarse-graining scheme over two stochastic coordinates measured from equilibrium simulations: the average energy $\langle E^* \rangle$ and the average energy trend $\langle t^* \rangle$, computed for each segment within the set Ω_i^{eq}. These two coordinates span the reduced stochastic landscape from which MSM states are inferred. Segments of data for which self-assembly has already occurred (indicated by an assembly flag equal to 1) are excluded from the coarse-graining and are instead assigned to a designated absorbing state, labeled FA (first assembly). This state acts as a sink in the MSM and is treated separately in the model construction.

We perform quantile-based binning on both stochastic coordinates to discretize the phase space. Let n_{bins} be the number of bins used for each coordinate. This value is chosen as: $n_{\mathrm{bins}} = \sqrt{N_{\mathrm{s}} - 1}$, where N_{s} is the total number of MSM states, including the FA sink state. To ensure symmetry and the presence of a central bin along the trend coordinate, a feature important for comparison with the nonequilibrium driven MSM, we constrain n_{bins} to be an odd integer.

Binning is performed as follows. First, all values of a stochastic coordinate (either energy or trend) are sorted. The smallest difference between adjacent values is defined as the jump size. We center two vertical lines symmetrically around the median value and iteratively expand them in steps equal to the jump size until the number of points within the resulting interval exceeds $|\Omega_i^{\mathrm{eq}}|/n_{\mathrm{bins}}$. This interval forms the central bin. New bins are then constructed outward from the central bin in both directions. At each step, a candidate bin edge is placed one jump size away from the current boundary. If the resulting bin contains a sufficient number of points (again exceeding $|\Omega_i^{\mathrm{eq}}|/n_{\mathrm{bins}}$), it is accepted, and otherwise, the bin is incrementally widened until it meets the criterion. This procedure continues until

exactly n_{bins} bins are created. The leftmost and rightmost bin edges are extended to $-\infty$ and ∞, respectively, to allow for future generalization of the model beyond the training data set minimal and maximal values. The bin edges for each coordinate are stored in a corresponding bin vector.

Each bin is assigned a natural number index, from 1 to n_{bins}, ordered from left to right. The average coordinate value within each bin represents the corresponding coarse-grained bin value. The whole state space of the MSM, without the FA state, is then constructed as the Cartesian product of the binned energy and trend coordinates, yielding n_{bins}^2 discrete states. Each state corresponds to a unique pair of bin indices: $k = 1, \ldots, n_{\text{bins}}$ for the energy coordinate and $m = 1, \ldots, n_{\text{bins}}$ for the trend coordinate. These indices label the coarse-grained average values $\langle E^* \rangle_k$ and $\langle t^* \rangle_m$ associated with each state.

Finally, each segment in Ω_i^{eq} is assigned to one of these coarse-grained states. If the assembly flag indicates completion, the segment is mapped directly to the FA state. Otherwise, its values of $\langle E^* \rangle$ and $\langle t^* \rangle$ are used to determine which bins they fall into, resulting in a state label determined by the pair of bin indices of $(\langle E^* \rangle_k, \langle t^* \rangle_m)$. This process generates a new coarse-grained data set where each segment is labeled with a corresponding $(\langle E^* \rangle_k, \langle t^* \rangle_m)$, and a state number of $n_{\text{bins}} \cdot (m-1) + k$. These numbers are used for the remaining process of the MSM construction. Examples of the states labeling and numbering appear in Tables S3 and S4.

S3.2 Constructing the Transition Matrix and Dwell Times Vector

The MSM transition matrix is constructed by iterating over the segments within each trajectory subset. First, a transition count matrix and a dwell time accumulator array are initialized with zeros. For each pair of consecutive segments, the current segment's state number and dwell time are recorded in the accumulator array. Simultaneously, a transition is registered from the current segment's state to the next by incrementing the corresponding entry in the transition count matrix.

Once all segments have been processed, the transition matrix is obtained by normalizing each row of the transition count matrix by the total number of outgoing transitions from that state, ensuring that each row sums to one and the matrix defines a valid set of transition probabilities. To ensure statistical robustness of the estimated transitions, we enforce a minimal sampling criterion, as described in Subsection S2.5 of the supporting information. The characteristic dwell time vector is computed by averaging the accumulated dwell times for each state. These mean dwell times define the exponential waiting time distributions assumed in the continuous-time MSM framework.

S3.3 Simulating the MSM

To generate an ensemble of coarse-grained stochastic trajectories, we simulate the constructed MSM. Each trajectory represents a potential assembly path through the coarse-grained state space, and its outcome is determined by whether it reaches the designated self-assembled FA sink state and when.

Each simulation begins by selecting an initial state at random, based on the measured initial state vector. The system then dwells in that state for a time drawn from an exponential distribution whose mean matches the state's characteristic residence time. Following this, the system transitions to a new state, randomly chosen according to the MSM's transition probability matrix. This process of sampling dwelling times and making state transitions is iterated over time. For each trajectory, we track the cumulative simulation time, the number of state transitions, and whether the trajectory reaches the self-assembled state. If the FA state is reached, the trajectory is terminated and labeled as successful, and the time-to-first-assembly (T_{FAS}) is recorded. If the trajectory exceeds a predefined simulation time limit or the maximum number of allowed steps, defined as the termination conditions, it is terminated and labeled as unsuccessful.

To set these termination conditions, we define a maximal MSM simulation time based on the median first-assembly time of the unassembled trajectories in the training set Ω_i^{eq}.

Similarly, we impose a step-count cutoff based on the median number of time segments observed in those unassembled trajectories. If all training trajectories reach assembly, these quantities are computed from the assembled subset instead. Across all simulated trajectories, we generate a corresponding array of binary assembly flags to record whether each trajectory reaches the FA state (assigned a value of 1) or not (assigned 0). This MSM ensemble provides the basis for calculating the observable quantities of the distribution of T_{FAS} and overall assembly success rates, $\%SA$.

S3.4 Constructing the Level-Jumping Matrices

The forward (T_{F}) and backward (T_{B}) level-jumping matrices capture the stochastic transitions between MSM states across drive activation and deactivation events. To construct these matrices, we begin by initializing both T_{F} and T_{B} as zero matrices of size $N_s \times N_s$, where N_s is the number of coarse-grained states. We then process the full set of driven trajectories contained in Ω^{neq}. For each drive-on event, we identify the segment state immediately preceding and immediately following the transition. We increment the corresponding element in T_{F}, with the row index representing the pre-transition state and the column index representing the post-transition state. An analogous procedure is applied for drive-off events to populate T_{B}.

After tallying all such transitions across the data set, both matrices are row-normalized to yield valid probability transition matrices. These normalized matrices represent the likelihood of transitioning between states across drive cycles and constitute the inter-layer coupling between the equilibrium MSMs of configurations 1 and 2. To ensure the statistical reliability of these transitions, we apply the minimal sampling condition defined in Subsection S2.5. In particular, we require that each row in T_{F} and, where feasible, in T_{B} is supported by at least n_{bound} transition events. This safeguards the robustness of the level-jumping transitions used in constructing the driven MSM.

S3.5 MSM-Driven Simulation Under Nonequilibrium Forcing

The nonequilibrium MSM-driven simulation begins in level 1 of the driven MSM (see main text), with an initial state sampled from the empirical state distribution of equilibrium configuration $i = 1$. The system then alternates between intra-layer MSM dynamics and inter-layer transitions that mimic the external driving protocol. The system dynamics undergoes the same process as in equilibrium, described in Subsection S3.3. The difference from the equilibrium case is that every $\tau_{\text{BEAST},i}$, a condition is checked, as described in the main text: if the system is found in a coarse-grained state with mean zero trend (highlighted in red in Figure 4 of the main text), an inter-layer transition is triggered.

This process of alternating intra-layer dynamics and conditional inter-layer transitions continues, with checks every $\tau_{\text{BEAST},i}$, until the simulation either reaches the absorbing FA state or exceeds the time or step limits defined by unassembled trajectories in Ω_1^{eq}. For each trajectory, the assembly flag and first assembly time T_{FAS} are recorded and used to compute the predicted yield ($\%SA$) and the T_{FAS} distribution. Kernel Density Estimation (KDE) is applied to evaluate the resulting distribution and assess the model's performance relative to the original driven simulations.

S4 Additional Results

This section presents supplementary results that support and extend the findings discussed in the main text. These results are either referenced directly in the main text or provide deeper insight into the model's performance and statistical reliability. The additional figures and analyses serve to further validate the construction and predictions of the MSM, both in the equilibrium and driven regimes.

S4.1 Implied Time Scale and Chapman–Kolmogorov Tests

As described in the main text, we examined memory in the coarse-grained dynamics using implied time scale (ITS) analysis and Chapman–Kolmogorov (CK) return-probability tests.[20] Configuration 1 (equilibrium coarse-graining with $N_{\mathrm{s}} = 10$ states, Table S3) was chosen for this examination. The base lag time τ_d was selected as described in the main text.

State trajectories were downsampled according to the chosen lag-time scheme, with initial samples trimmed as needed to align with multiples of τ_d. Within each updated subset Ω_1^{eq} (the stochastic-coordinate partition used to coarse-grain the system), we truncated each trajectory at its first-assembly event and accumulated transition counts among the non-absorbing states and into the absorbing state. We set the examined horizons to $t_{k'} = k' \tau_d$ for $k' = 0, \ldots, \lfloor 0.2/\tau_d \rfloor$ (i.e., up to 0.2 [s]), ensuring alignment to multiples of τ_d. The state-transition counts $C_{s'j'}(k'\tau_d)$ were then defined as

$$C_{s'j'}(k'\tau_d) \;=\; \#\{\text{segments of length } k'\tau_d \text{ that start in } s' \text{ and end in } j'\}\,. \tag{S.14}$$

Row normalization yields the empirical row-stochastic transition matrix, defined as

$$P_{s'j'}(k'\tau_d) = C_{s'j'}(k'\tau_d) \Big/ \sum_{j'} C_{s'j'}(k'\tau_d). \tag{S.15}$$

For $\tau \in [4 \times 10^{-6},\, 0.2]$ [s], we used 20 logarithmically spaced samples (zero excluded) and computed the eigenvalues $\{\lambda_{i'}(\tau)\}$ of $P(\tau)$. We then converted them to implied time scales via the standard relation

$$t_{i'}(\tau) \;=\; -\frac{\tau}{\ln \lambda_{i'}(\tau)}. \tag{S.16}$$

We plotted the five slowest $t_{i'}(\tau)$ against the model lag τ on a semilog ordinate (linear time on the abscissa) in Figure S6 (left panel). Diamonds, circles, and squares mark τ_d, $5\tau_d$,

and $10\tau_d$ possible lag-time choices, respectively. $5\tau_d$ and $10\tau_d$ were additionally considered as candidate lag times, to compare results against the base choice τ_d.

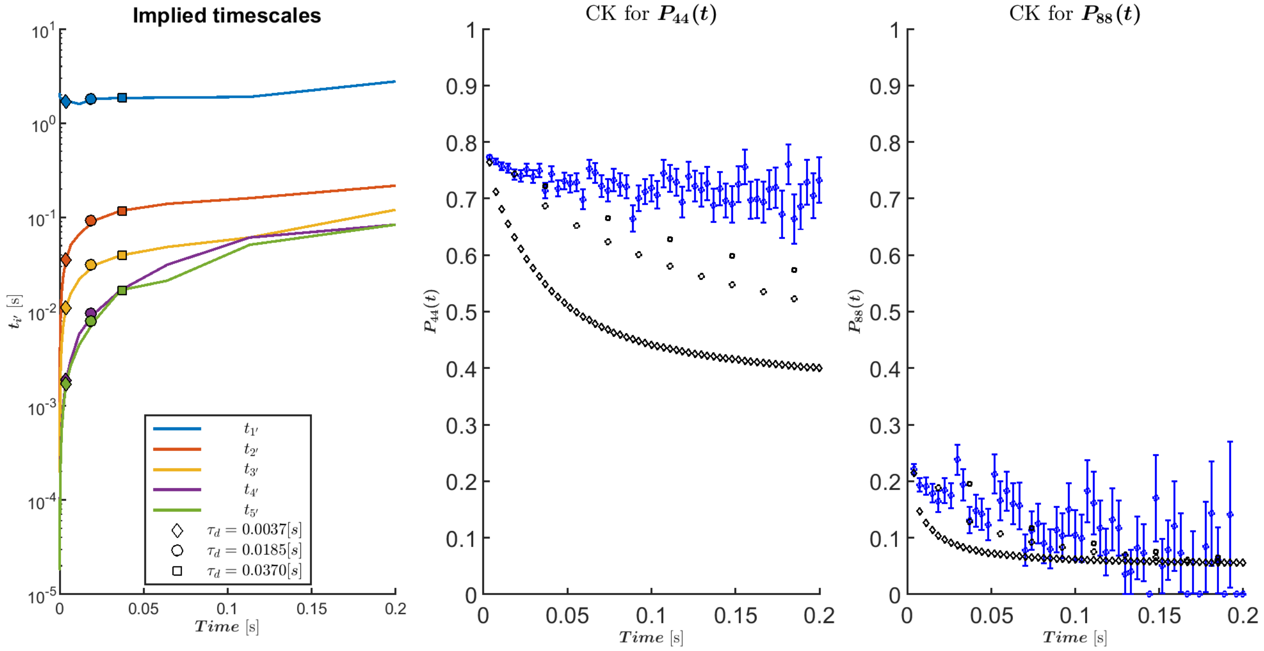

Figure S6: **Implied time scales and CK tests.** The left panel presents the slowest implied time scales from $P(\tau)$ for different times over non-sink states (semilog ordinate). Markers denote τ_d (diamond), $5\tau_d$ (circle), and $10\tau_d$ (square) lag-time choices. The middle and right panels respectively present the Chapman–Kolmogorov return probabilities for exemplar states $P_{44}(t)$ and $P_{88}(t)$: blue points with one-sigma bars are empirical estimates for $t_{k'} = k'\,\tau_d$ with $k' = 0, \ldots, \lfloor 0.2/\tau_d \rfloor$; black symbols are MSM predictions $[P(\tau_d)^{k'}]_{s's'}$, matching the lag-time selection symbols used in the left-panel legend, for states $s' = 4$ or $s' = 8$. Each has corresponding statistics and labels matching the state number in configuration 1 equilibrium MSM with $N_s = 10$ states, as in Table S3. Error bars follow Prinz *et al.*[20]

We now refer to the CK test. The smallest-lag transition matrix $P(\tau_d)$ was used to define the hypothetical Markov-state prediction $P_{\text{MSM}} = P(\tau_d)^{k'}$. We set $p_{\text{MSM}}(s', s'; k'\tau_d) = \left[P_{\text{MSM}}\right]_{s's'}$, i.e., the corresponding matrix element for state s'. We define $P_{s's'}(k'\tau_d)$ as the empirical return probability at $k'\tau_d$ for state s', calculated by Eq. (S.15). We then visually compare the empirical return probability with the hypothetical Markov prediction for states $s' = 4$ and $s' = 8$ in Figure S6 (middle and right panels); each has corresponding statistics and labels matching the state number in configuration 1 equilibrium MSM with $N_s = 10$ states, as in Table S3. These two states were chosen because they attain, respectively,

the largest implied time scale and a median implied time scale among the eigenvalues of $P(\tau = \tau_d)$. Uncertainty bands for empirical return probabilities were computed from count statistics following the construction recommended by Prinz *et al.*[20] and are plotted for each case in Figure S6 (middle and right panels, blue).

To quantify deviation from, or correspondence to, Markovianity with respect to the implied time scales in Figure S6 left panel, visual plateauing of $t_{i'}(\tau)$ with increasing τ indicates improved approximate Markovianity of those slow processes, whereas lack of plateauing signals residual memory not absorbed by the chosen lag;[20] With respect to the CK, the comparison is judged by whether the hypothetical MSM probabilities lie within the statistical uncertainty of the empirical probabilities (the error bars) over a range of lag times greater than the chosen base lag.

From the figure panels, we find that at the base lag τ_d, several ITS curves do not yet plateau and CK predictions conspicuously deviate from the empirical bands, indicating that the coarse-grained dynamics retain memory. Increasing τ to $5\tau_d$ and $10\tau_d$ moves CK predictions closer to the empirical uncertainty and produces more pronounced ITS plateaus, consistent with the known effect that larger lags reduce apparent memory.

It is important to emphasize that, for the examined lag times, Markovianity *per se* was not found for the KMC simulation, and further increasing the lag time would overlook first-assembly events occurring beneath that lag, which we refrain from, as we aim to capture the assembly yield and first-assembly-time distributions in this work. Although the underlying KMC dynamics exhibit memory at the chosen lag τ_d, we deliberately neglect this memory and construct an MSM with the same drawn statistics, labels, and dwell times, seeking to capture, with good precision, the KMC time-to-first-assembly distribution and yield despite this neglect.

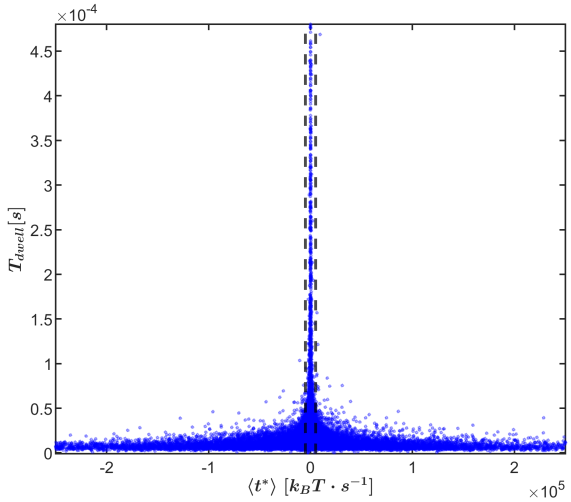

Figure S7: Dwell time as a function of mean trend for configuration 1. The relationship between the segment dwell time and the average energy trend is based on stochastic landscape segments obtained using the equilibrium simulation scheme described in Figure 2A of the main text. The interaction parameters for this configuration are $J_s = -3.6$ $[k_B T]$ and $J_w = -1$ $[k_B T]$, with all other simulation parameters provided in Table S1. The shaded region around zero trend indicates the kinetic trap domain (T^*), as defined in the Methods section of the main text. This region characterizes slow dynamics and serves as the criterion for triggering drive activation in the nonequilibrium protocol.

Table S3: Energy and trend bin assignments for the $N_s = 10$ MSMs. Representative mean energy $\langle E^* \rangle_k$ and mean trend $\langle t^* \rangle_m$, as described in the main text, for the equilibrium MSMs are shown vs. their state number, with respective energy (k) and trend (m) bin numbers. State 10 is the FA sink state, which is not included in the table.

**State**	(k, m)	$(\langle E^* \rangle_k, \langle t^* \rangle_m)$ **of** $i = 1$ **MSM**	$(\langle E^* \rangle_k, \langle t^* \rangle_m)$ **of** $i = 2$ **MSM**
1	$(1, 1)$	$(-131.3,\ -8.25 \times 10^3)$	$(-86.5,\ -2.75 \times 10^5)$
2	$(2, 1)$	$(-123.9,\ -8.25 \times 10^3)$	$(-68.6,\ -2.75 \times 10^5)$
3	$(3, 1)$	$(-115.9,\ -8.25 \times 10^3)$	$(-54.8,\ -2.75 \times 10^5)$
4	$(1, 2)$	$(-131.3,\ 14)$	$(-86.5,\ 950)$
5	$(2, 2)$	$(-123.9,\ 14)$	$(-68.6,\ 950)$
6	$(3, 2)$	$(-115.9,\ 14)$	$(-54.8,\ 950)$
7	$(1, 3)$	$(-131.3,\ 8.0 \times 10^3)$	$(-86.5,\ 2.75 \times 10^5)$
8	$(2, 3)$	$(-123.9,\ 8.0 \times 10^3)$	$(-68.6,\ 2.75 \times 10^5)$
9	$(3, 3)$	$(-115.9,\ 8.0 \times 10^3)$	$(-54.8,\ 2.75 \times 10^5)$

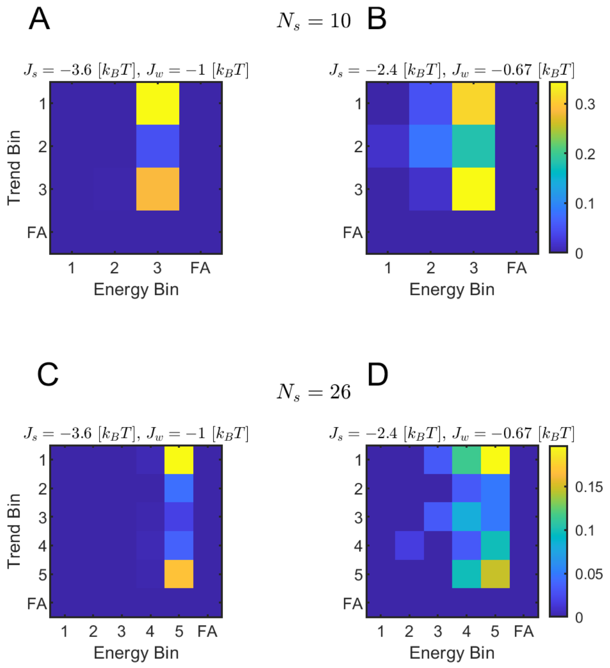

Figure S8: The MSM initial states heatmaps for configurations 1 and 2. Initial state probability distributions for two configurations: configuration 1 (left; panels A and C) and configuration 2 (right; panels B and D) are presented. Panels A and B correspond to $N_\mathrm{s} = 10$ states, while panels C and D correspond to $N_\mathrm{s} = 26$ states. The heatmaps represent the initial probabilities across energy and trend bins, normalized so that each map sums to 1. Bin definitions for energy and trend are provided in Tables S3 and S4, respectively.

Table S4: Energy and trend bin assignments for the $N_\mathrm{s} = 26$ MSMs. Representative mean energy $\langle E^* \rangle_k$ and mean trend $\langle t^* \rangle_m$, as described in the main text, for the equilibrium MSMs are shown vs. their state number, with respective energy (k) and trend (m) bin numbers. State 26 is the FA sink state, which is not included in the table.

**State**	(k, m)	$(\langle E^* \rangle_k, \langle t^* \rangle_m)$ **of** $i = 1$ **MSM**	$(\langle E^* \rangle_k, \langle t^* \rangle_m)$ **of** $i = 2$ **MSM**
1	$(1, 1)$	$(-132.7,\ -1.95 \times 10^4)$	$(-87.6,\ -4.5 \times 10^5)$
2	$(2, 1)$	$(-127.8,\ -1.95 \times 10^4)$	$(-80.4,\ -4.5 \times 10^5)$
3	$(3, 1)$	$(-123.9,\ -1.95 \times 10^4)$	$(-68.5,\ -4.5 \times 10^5)$
4	$(4, 1)$	$(-119.7,\ -1.95 \times 10^4)$	$(-60.3,\ -4.5 \times 10^5)$
5	$(5, 1)$	$(-112.4,\ -1.95 \times 10^4)$	$(-51.4,\ -4.5 \times 10^5)$
6	$(1, 2)$	$(-132.7,\ -1.525 \times 10^3)$	$(-87.6,\ -9.0 \times 10^4)$
7	$(2, 2)$	$(-127.8,\ -1.525 \times 10^3)$	$(-80.4,\ -9.0 \times 10^4)$
8	$(3, 2)$	$(-123.9,\ -1.525 \times 10^3)$	$(-68.5,\ -9.0 \times 10^4)$
9	$(4, 2)$	$(-119.7,\ -1.525 \times 10^3)$	$(-60.3,\ -9.0 \times 10^4)$
10	$(5, 2)$	$(-112.4,\ -1.525 \times 10^3)$	$(-51.4,\ -9.0 \times 10^4)$
11	$(1, 3)$	$(-132.7,\ 1.625 \times 10^1)$	$(-87.6,\ 1.75 \times 10^3)$
12	$(2, 3)$	$(-127.8,\ 1.625 \times 10^1)$	$(-80.4,\ 1.75 \times 10^3)$
13	$(3, 3)$	$(-123.9,\ 1.625 \times 10^1)$	$(-68.5,\ 1.75 \times 10^3)$
14	$(4, 3)$	$(-119.7,\ 1.625 \times 10^1)$	$(-60.3,\ 1.75 \times 10^3)$
15	$(5, 3)$	$(-112.4,\ 1.625 \times 10^1)$	$(-51.4,\ 1.75 \times 10^3)$
16	$(1, 4)$	$(-132.7,\ 1.675 \times 10^3)$	$(-87.6,\ 9.5 \times 10^4)$
17	$(2, 4)$	$(-127.8,\ 1.675 \times 10^3)$	$(-80.4,\ 9.5 \times 10^4)$
18	$(3, 4)$	$(-123.9,\ 1.675 \times 10^3)$	$(-68.5,\ 9.5 \times 10^4)$
19	$(4, 4)$	$(-119.7,\ 1.675 \times 10^3)$	$(-60.3,\ 9.5 \times 10^4)$
20	$(5, 4)$	$(-112.4,\ 1.675 \times 10^3)$	$(-51.4,\ 9.5 \times 10^4)$
21	$(1, 5)$	$(-132.7,\ 1.9 \times 10^4)$	$(-87.6,\ 4.75 \times 10^5)$
22	$(2, 5)$	$(-127.8,\ 1.9 \times 10^4)$	$(-80.4,\ 4.75 \times 10^5)$
23	$(3, 5)$	$(-123.9,\ 1.9 \times 10^4)$	$(-68.5,\ 4.75 \times 10^5)$
24	$(4, 5)$	$(-119.7,\ 1.9 \times 10^4)$	$(-60.3,\ 4.75 \times 10^5)$
25	$(5, 5)$	$(-112.4,\ 1.9 \times 10^4)$	$(-51.4,\ 4.75 \times 10^5)$

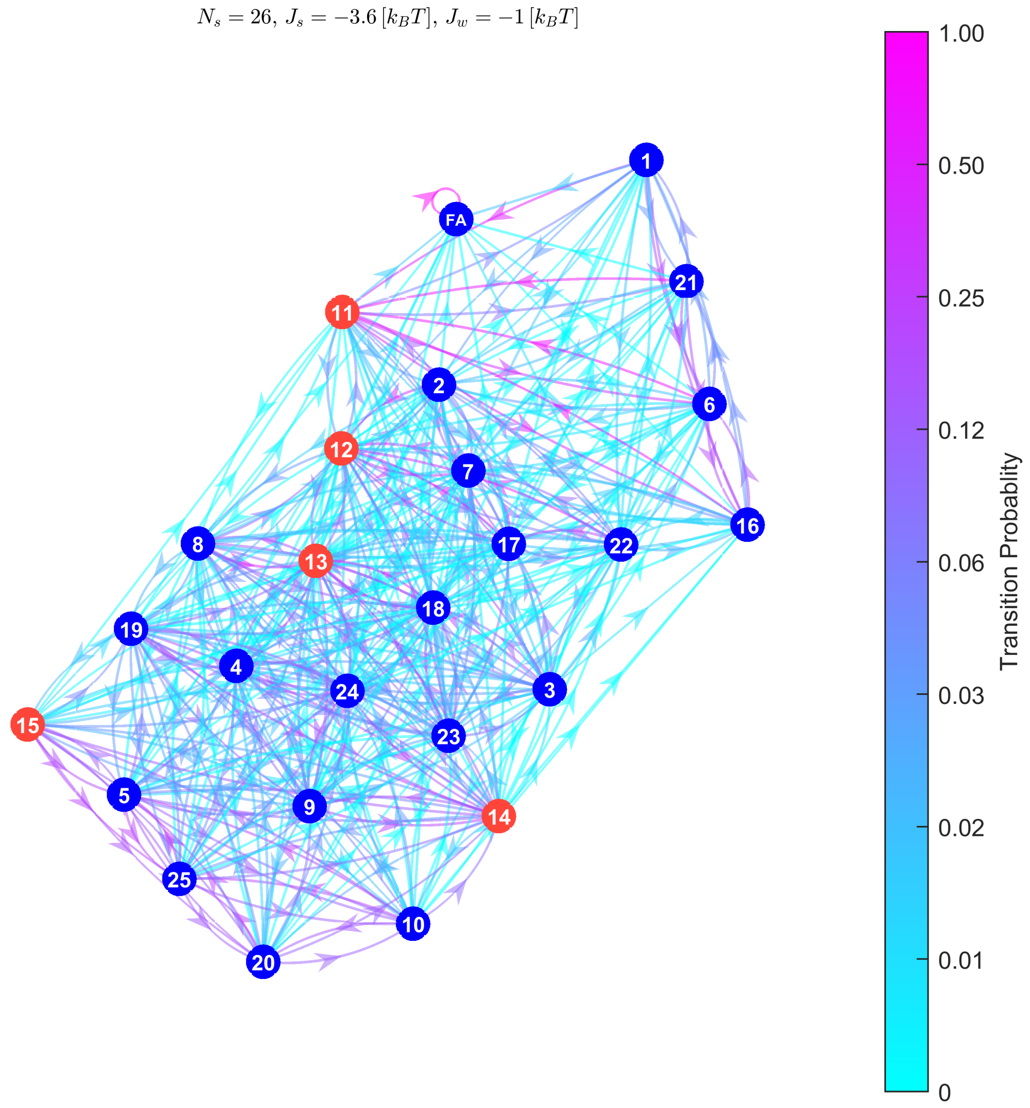

Figure S9: Configuration 1 equilibrium MSM with $N_s = 26$ states. Each state is labeled by its corresponding state number, representing its mean energy and mean trend values as listed in Table S4. Red-colored nodes indicate states that activate the control drive in the scheme described in Figure 2C. This equilibrium MSM serves as the level-1 layer, as described in the main text. Directed edges represent transition probabilities between states. The node labeled 'FA' denotes the first assembly event sink.

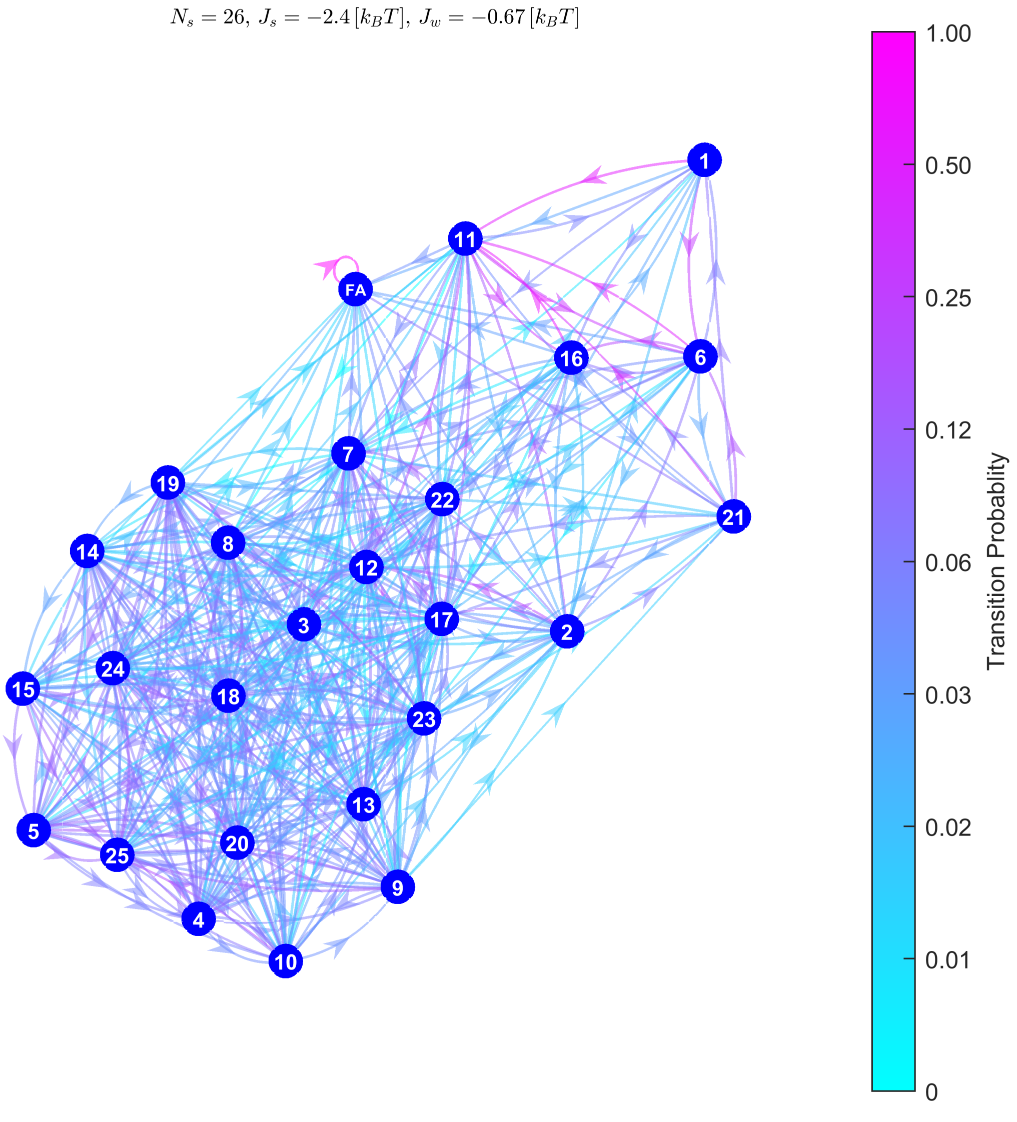

Figure S10: Configuration 2 equilibrium MSM with $N_\mathrm{s} = 26$ states. Each state is labeled by its corresponding state number, representing its mean energy and mean trend values as listed in Table S4. In the driven scheme of Figure 2C of the main text, we use this equilibrium MSM as the level 2 layer, as described in the main text. Directed edges represent transition probabilities between states. The node labeled 'FA' denotes the first assembly event sink.

S4.2 Representative States Snapshots

Figure S11 shows representative snapshots from the ten-state MSM under the default configuration. States in the same row share the same coarse-grained (CG) mean trend $\langle t^* \rangle$, whereas states in the same column share the same CG mean energy $\langle E^* \rangle$. As energy decreases across columns, strong bonds, depicted with a marker between two adjacent particles edges, generally become more frequent and the largest cluster becomes more compact (lower perimeter-to-area ratio), approaching the square targets stored in memory (state 10).

Interpreting variation with trend is less direct from a single frame, but the third column, where fixed weak binding energy is present and the trend varies by row, is illustrative. The negative trend, near-zero trend, and positive trend correspond to the first, second, and third rows, respectively. A slightly negative trend is observed in several smaller clusters, characterized by transient breakups that lower the energy and promote coalescence into a single cluster, resulting in an average energy decrease in these macrostates. A near-zero trend indicates a compact cluster with a low perimeter-to-area ratio; escape events are rare, so the system remains stable for long periods as particles detach and reattach, yielding a long-lived kinetic trap. A positive trend reveals a large cluster with an unstable protrusion; the system tends to shed this protrusion until a stable, more compact cluster remains, producing a positive mean energy trend en route to that endpoint.

In the first column (lowest energy), differences across trends reflect internal state flips and local rearrangements within clusters that strengthen nearest-neighbor bonds, rather than large-scale aggregation or breakup.

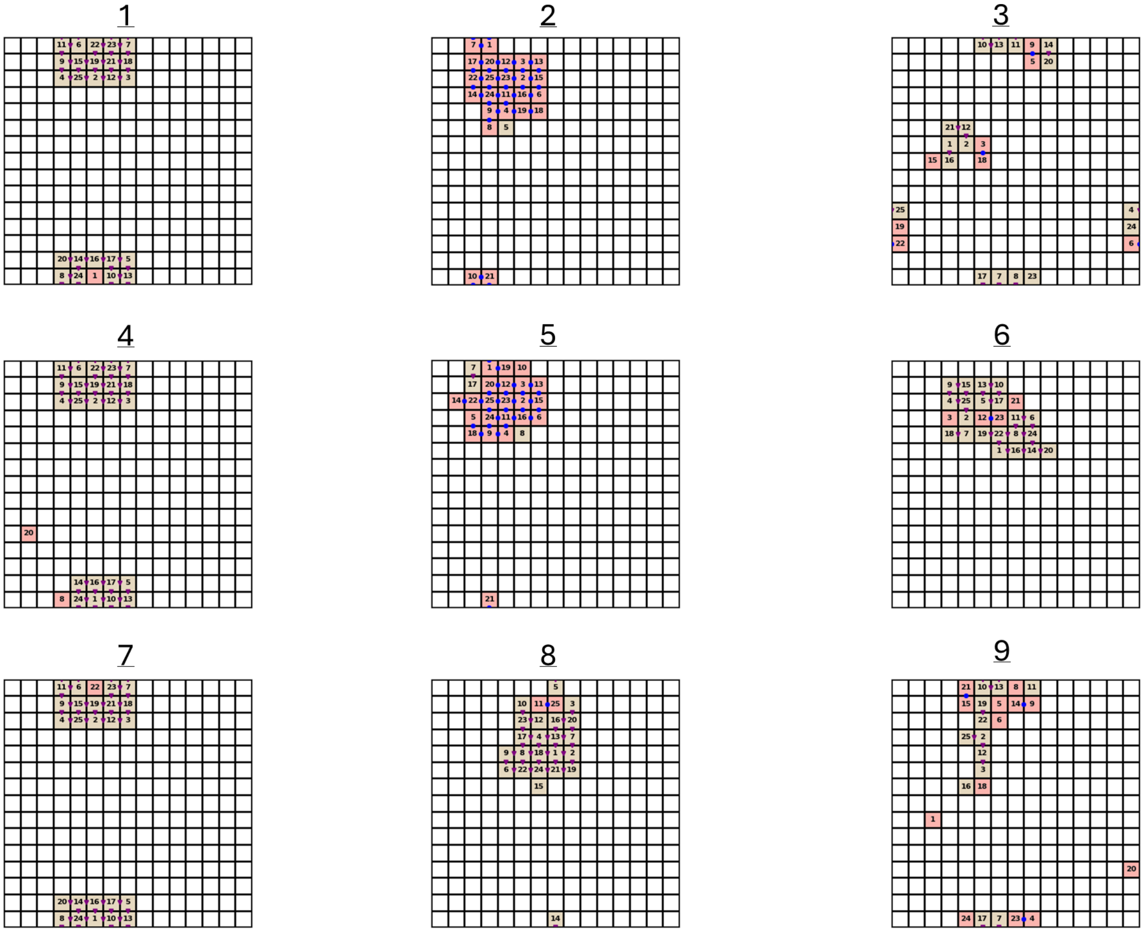

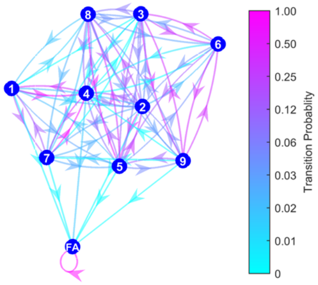

State	(k, m)	$(\langle E^* \rangle_k, \langle t^* \rangle_m)$ **of** $i = 1$ **MSM**
1	$(1, 1)$	$(-131.3,\ -8.25 \times 10^3)$
2	$(2, 1)$	$(-123.9,\ -8.25 \times 10^3)$
3	$(3, 1)$	$(-115.9,\ -8.25 \times 10^3)$
4	$(1, 2)$	$(-131.3,\ 14)$
5	$(2, 2)$	$(-123.9,\ 14)$
6	$(3, 2)$	$(-115.9,\ 14)$
7	$(1, 3)$	$(-131.3,\ 8.0 \times 10^3)$
8	$(2, 3)$	$(-123.9,\ 8.0 \times 10^3)$
9	$(3, 3)$	$(-115.9,\ 8.0 \times 10^3)$

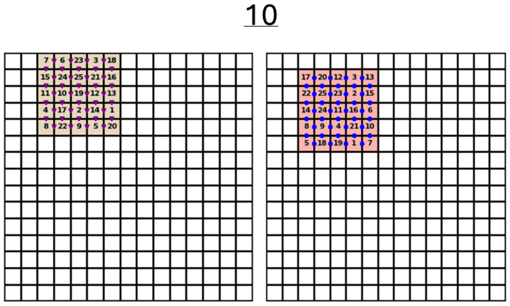

Figure S11: Coarse-grained state snapshots for the ten-state MSM under the default configuration. Snapshots were extracted from distinct KMC trajectories while the system dwelled in the segments corresponding to each coarse-grained state. State 10 corresponds to assembly of either of the two stored targets in the lattice. State indices match those in Table S3 (left column) and the MSM shown in Figure 4. For clarity, both the table and the MSM are reproduced in the bottom row of this figure.

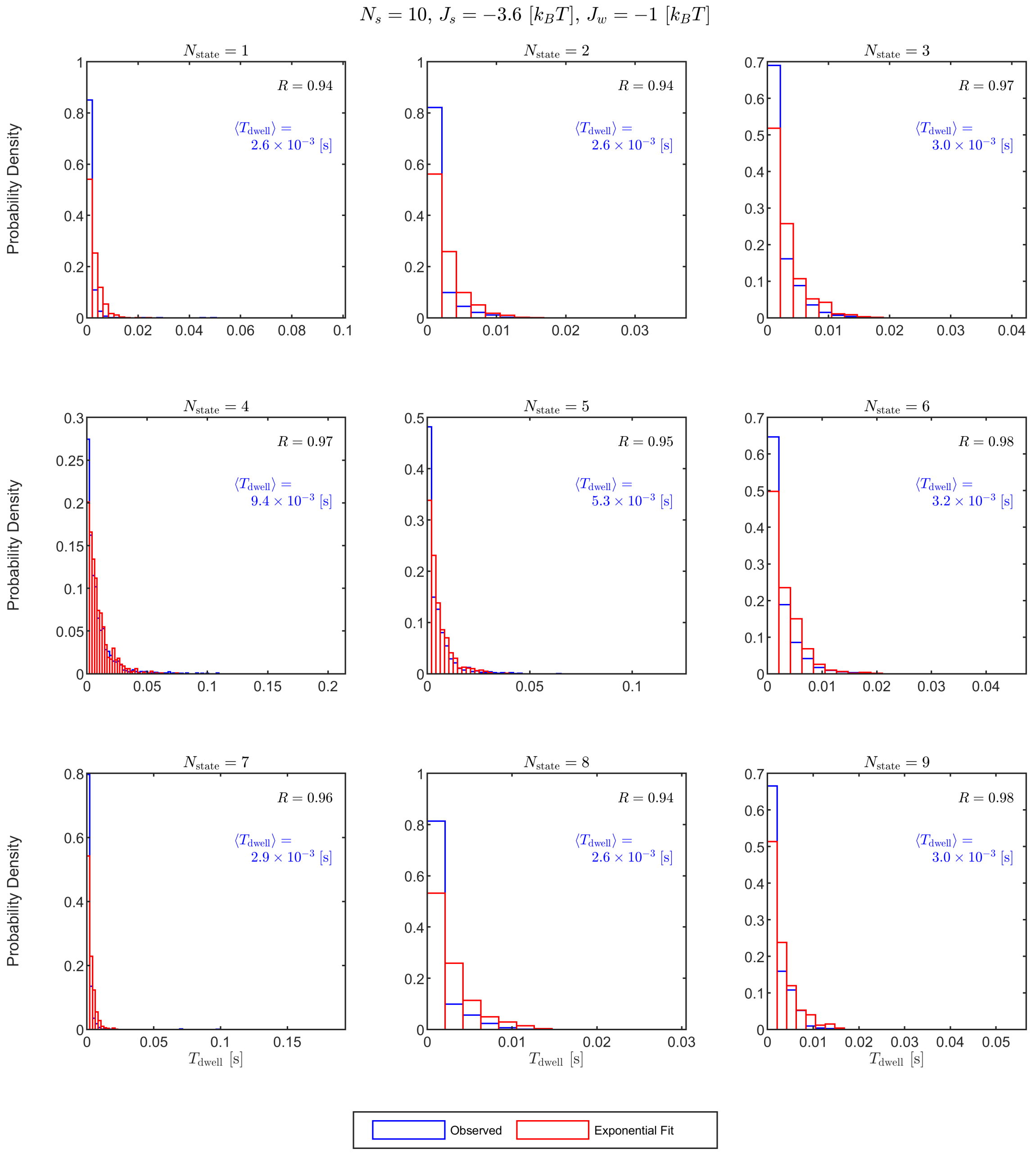

Figure S12: Dwell time distributions of equilibrium MSM states for configuration 1 with $N_\text{s} = 10$ coarse-grained states. Each panel displays a histogram of dwell times for one of the MSM states, based on the equilibrium simulation of configuration 1. The dwell time histograms (blue) are constructed using Scott's rule for bin width selection. For comparison, an exponential distribution (red) is generated with the same mean dwell time and binned using identical bin edges. The Pearson correlation coefficient R is reported in each panel, quantifying the agreement between the observed dwell time distribution and the exponential model. This comparison assesses how well each coarse-grained state conforms to the continuous-time Markov assumption, where dwell times are expected to follow an exponential distribution. State indices correspond to those listed in Table S3.

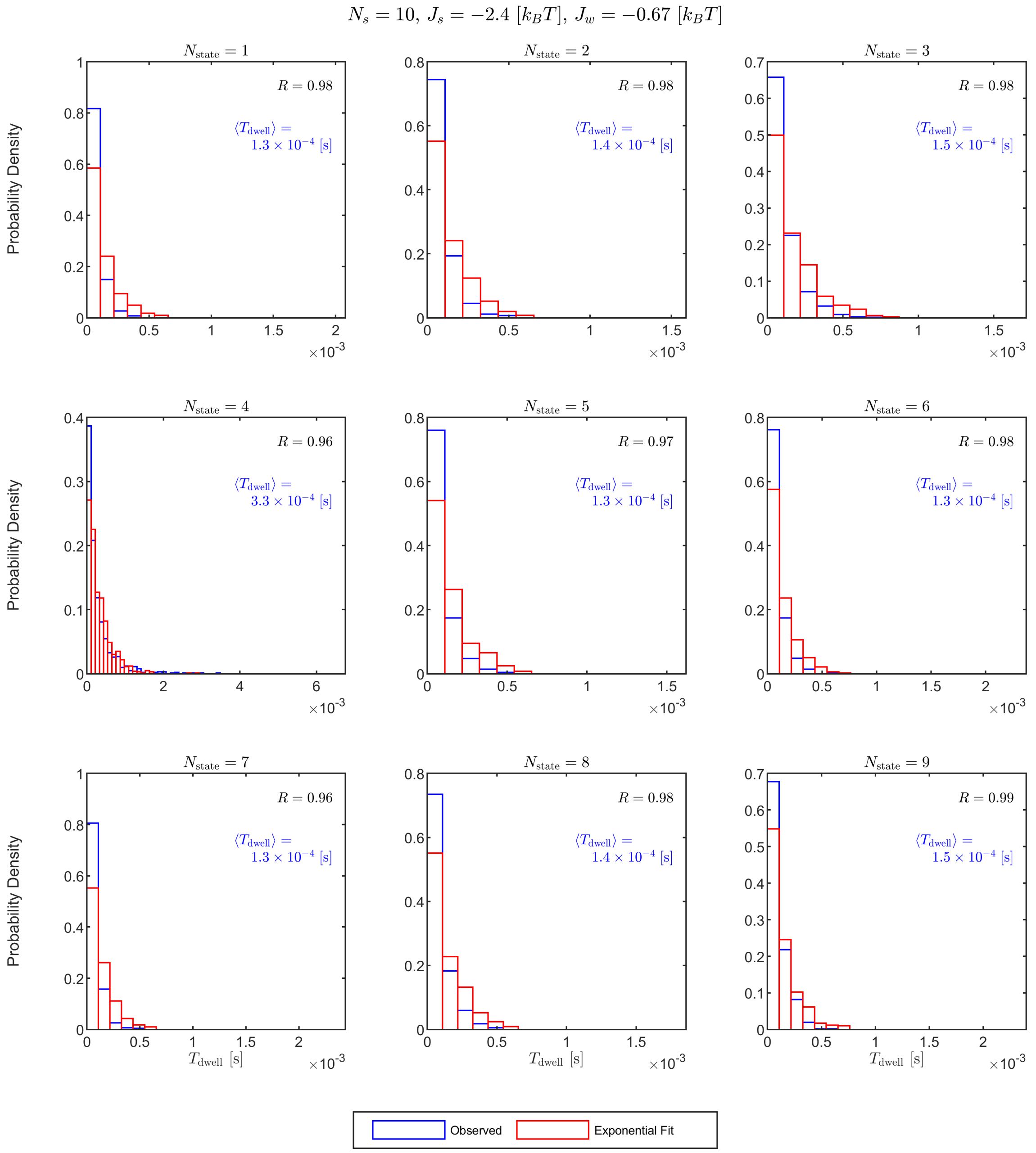

Figure S13: Dwell time distributions of equilibrium MSM states for configuration 2 with $N_{\mathrm{s}} = 10$ coarse-grained states. Each panel displays a histogram of dwell times for one of the MSM states, based on the equilibrium simulation of configuration 2. The dwell time histograms (blue) are constructed using Scott's rule for bin width selection. For comparison, an exponential distribution (red) is generated with the same mean dwell time and binned using identical bin edges. The Pearson correlation coefficient R is reported in each panel, quantifying the agreement between the observed dwell time distribution and the exponential model. This comparison assesses how well each coarse-grained state conforms to the continuous-time Markov assumption, where dwell times are expected to follow an exponential distribution. State indices correspond to those listed in Table S3.

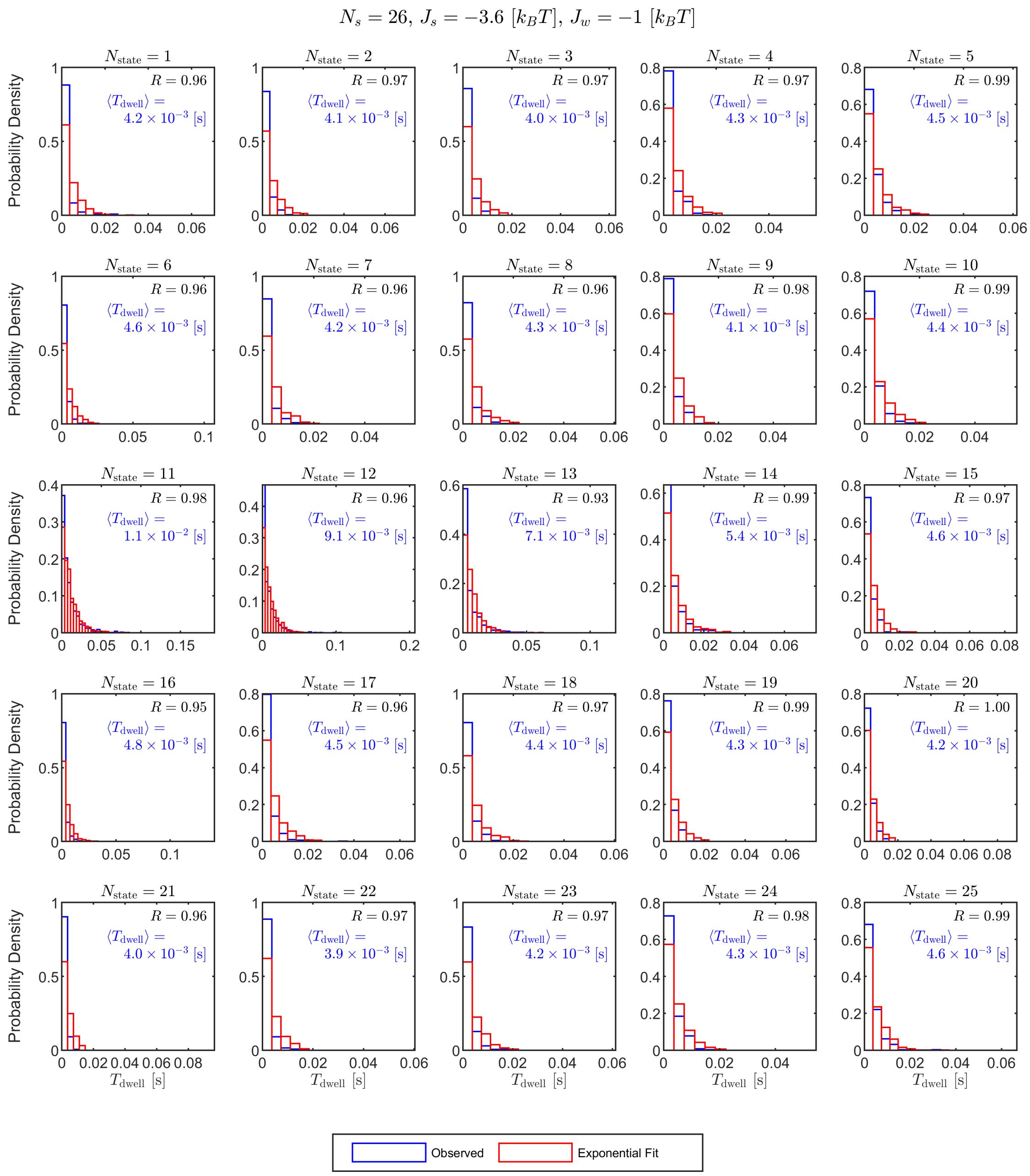

Figure S14: Dwell time distributions of equilibrium MSM states for configuration 1 with $N_s = 26$ coarse-grained states. Each panel displays a histogram of dwell times for one of the MSM states, based on the equilibrium simulation of configuration 1. The dwell time histograms (blue) are constructed using Scott's rule for bin width selection. For comparison, an exponential distribution (red) is generated with the same mean dwell time and binned using identical bin edges. The Pearson correlation coefficient R is reported in each panel, quantifying the agreement between the observed dwell time distribution and the exponential model. This comparison assesses how well each coarse-grained state conforms to the continuous-time Markov assumption, where dwell times are expected to follow an exponential distribution. State indices correspond to those listed in Table S4.

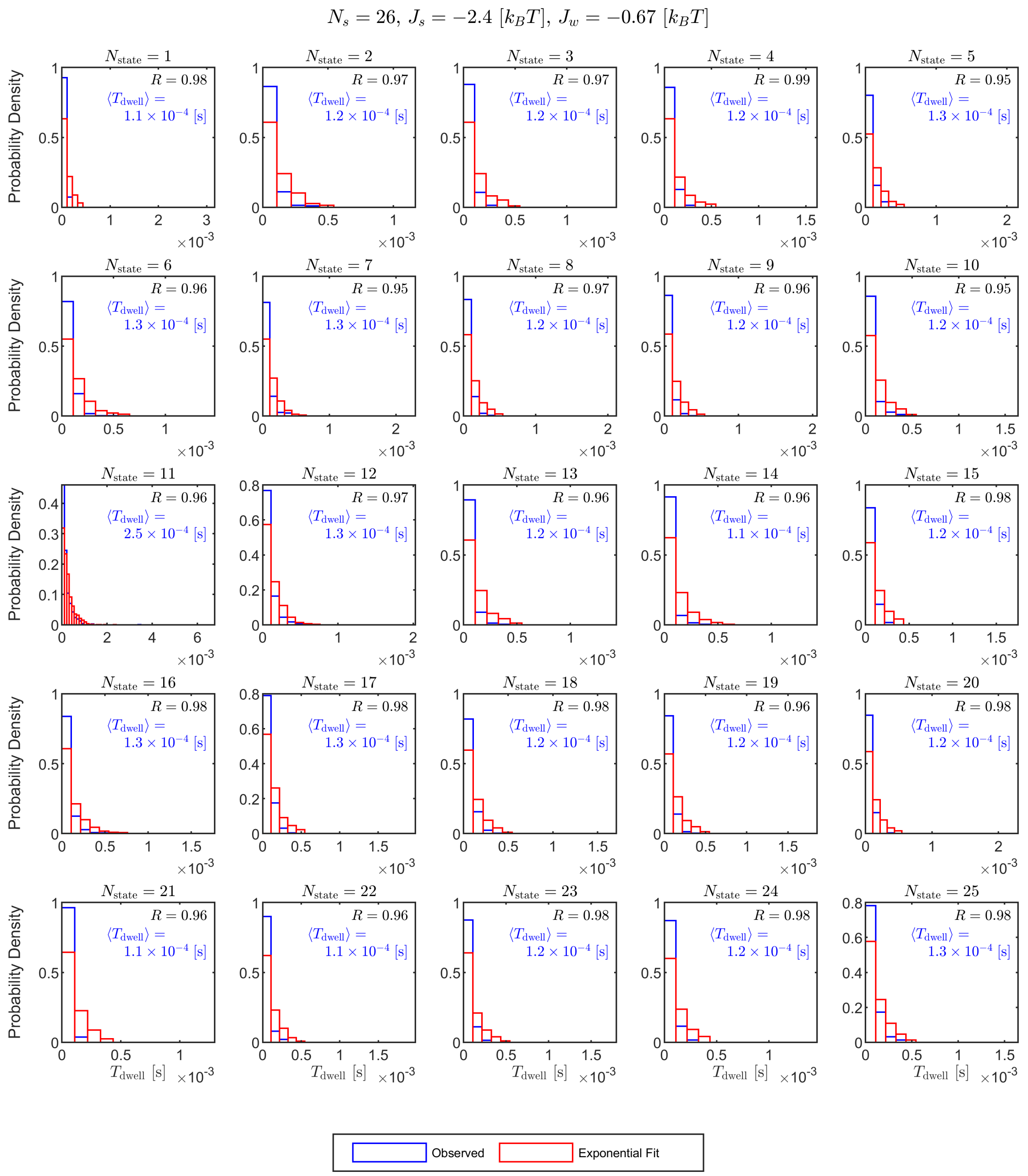

Figure S15: Dwell time distributions of equilibrium MSM states for configuration 2 with $N_{\text{s}} = 26$ coarse-grained states. Each panel displays a histogram of dwell times for one of the MSM states, based on the equilibrium simulation of configuration 2. The dwell time histograms (blue) are constructed using Scott's rule for bin width selection. For comparison, an exponential distribution (red) is generated with the same mean dwell time and binned using identical bin edges. The Pearson correlation coefficient R is reported in each panel, quantifying the agreement between the observed dwell time distribution and the exponential model. This comparison assesses how well each coarse-grained state conforms to the continuous-time Markov assumption, where dwell times are expected to follow an exponential distribution. State indices correspond to those listed in Table S4.

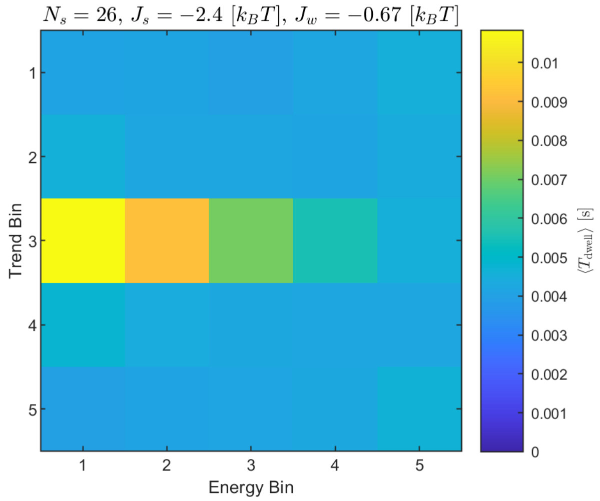

Figure S16: $\langle T_{\mathrm{dwell}} \rangle$ vs. state index for configuration 2 equilibrium MSM with $N_{\mathrm{s}} = 26$ states. $\langle T_{\mathrm{dwell}} \rangle$ is plotted against the corresponding (k, m) bin indices as defined in Table S4.

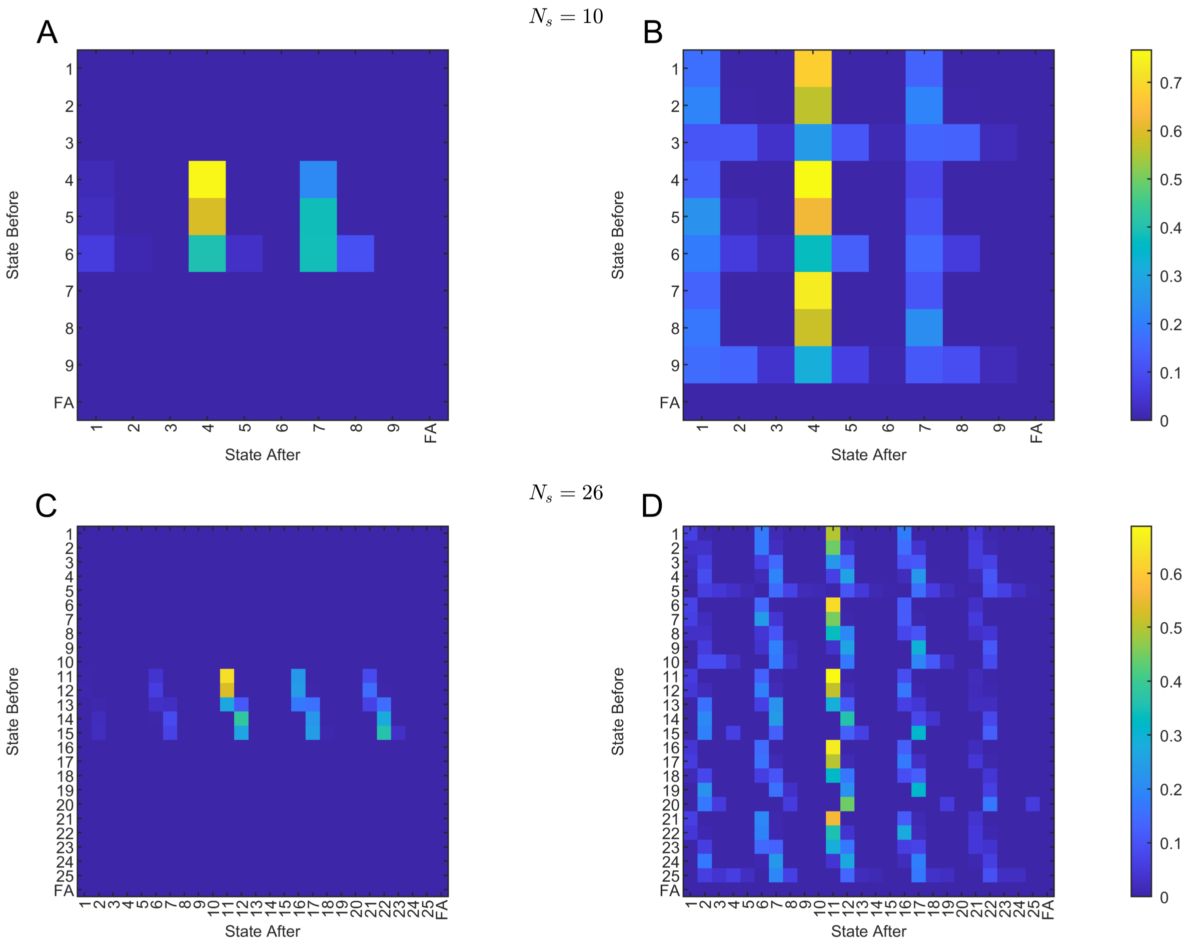

Figure S17: Forward (T_{F}) and backward (T_{B}) transition matrices for the driven MSMs. T_{F} and T_{B} are plotted left and right, respectively, for $N_{\mathrm{s}} = 10$ (top row, panels A–B) and $N_{\mathrm{s}} = 26$ (bottom row, panels C–D). The matrices were obtained from the Ω^{neq} data (with $\tau_{\mathrm{L}}^{\mathrm{neq}}$ shock time) by gathering forward transitions from configuration 1 to 2, and backward transitions from 2 to 1 respectively, as described in the main text. All matrices were normalized row-wise to yield transition probability vectors, given the state before the transition, and visualized on a consistent color scale within each row. The row and column matrices' numbers represent the state numbers of Table S3 for $N_{\mathrm{s}} = 10$ and of Table S4 $N_{\mathrm{s}} = 26$, with the final state marked as 'FA'.

S5 Computational Benchmarking

To assess the computational advantages of the MSM framework, we conducted a series of benchmarking tests comparing its runtime to that of direct KMC simulations. All simulations were executed on the same hardware platform: an Intel(R) Core(TM) i9-10920X CPU operating at 3.50 GHz with 32 GB of RAM, running Windows 11. The KMC simulations were implemented in Python using the PyCharm environment, while MSM computations were carried out in MATLAB R2025a.

Benchmarking focused on the average computation time required to generate a single simulation trajectory. Both equilibrium and driven assembly scenarios were tested using the coarse-grained models with 26 discrete states. For the driven case, the evaluation included all tested values of the drive activation duration, and runtimes were averaged across these conditions. Each KMC result reflects the mean of 60 trajectories, while MSM performance was averaged over 1000 realizations.

We found that the runtime of the driven KMC simulations was largely insensitive to the specific drive duration used. On average, KMC simulations required approximately 5 hours (18 million milliseconds) for equilibrium assembly and 8 hours (28.8 million milliseconds) for driven assembly. In stark contrast, the corresponding MSM simulations completed in only 0.32 milliseconds (equilibrium) and 3.7 milliseconds (driven), despite the use of a larger ensemble.

These results demonstrate a computational acceleration of approximately seven to eight orders of magnitude when using the MSM framework in place of KMC simulations. This dramatic reduction in runtime, coupled with the model's ability to retain accuracy, underscores the value of the MSM as an efficient surrogate for exploring and optimizing nonequilibrium self-assembly protocols.

References

(1) Bortz, A. B.; Kalos, M. H.; Lebowitz, J. L. A new algorithm for Monte Carlo simulation of Ising spin systems. *J. Comp. Phys.* **1975**, *17*, 10–18.

(2) Gillespie, D. T. A general method for numerically simulating the stochastic time evolution of coupled chemical reactions. *J. Comp. Phys.* **1976**, *22*, 403–434.

(3) Bisker, G.; England, J. L. Nonequilibrium associative retrieval of multiple stored self-assembly targets. *Proc. Natl. Acad. Sci. U. S. A.* **2018**, *115*, E10531–E10538.

(4) Faran, M.; Bisker, G. Nonequilibrium self-assembly time forecasting by the stochastic landscape method. *J. Phys. Chem. B* **2023**, *127*, 6113–6124.

(5) Newman, M. E.; Barkema, G. T. *Monte Carlo methods in statistical physics*; Clarendon Press, 1999.

(6) Choe, Y.; Magnasco, M. O.; Hudspeth, A. A model for amplification of hair-bundle motion by cyclical binding of Ca2+ to mechanoelectrical-transduction channels. *Proc. Natl. Acad. Sci. U. S. A.* **1998**, *95*, 15321–15326.

(7) Qian, H. Open-system nonequilibrium steady state: statistical thermodynamics, fluctuations, and chemical oscillations. *J. Phys. Chem. B* **2006**, *110*, 15063–15074.

(8) Straub, J. E. Analysis of the role of attractive forces in self-diffusion of a simple fluid. *Mol. Phys.* **1992**, *76*, 373–385.

(9) Whitelam, S.; Geissler, P. L. Avoiding unphysical kinetic traps in Monte Carlo simulations of strongly attractive particles. *J. Chem. Phys.* **2007**, *127*, 154101.

(10) Zhao, K.; Wulder, M. A.; Hu, T.; Bright, R.; Wu, Q.; Qin, H.; Li, Y.; Toman, E.; Mallick, B.; Zhang, X. e. a. Detecting change-point, trend, and seasonality in satellite time series data to track abrupt changes and nonlinear dynamics: A Bayesian ensemble algorithm. *Rem. Sen. Environ.* **2019**, *232*, 111181.

(11) Faran, M.; Ray, D.; Nag, S.; Raucci, U.; Parrinello, M.; Bisker, G. A stochastic landscape approach for protein folding state classification. *J. Chem. Theory and Comput.* **2024**, *20*, 5428–5438, PMID: 38924770.

(12) Faran, M.; Bisker, G. Nonequilibrium self-assembly control by the stochastic landscape method. *J. Chem. Inf. Model.* **2025**, *65*, 4067–4080.

(13) Smilgies, D.-M.; Folta-Stogniew, E. Molecular weight–gyration radius relation of globular proteins: a comparison of light scattering, small-angle X-ray scattering and structure-based data. *J. Appl. Cryst.* **2015**, *48*, 1604–1606.

(14) Dix, J. A.; Verkman, A. Crowding effects on diffusion in solutions and cells. *Ann. Rev. Biophys.* **2008**, *37*, 247–263.

(15) Metzler, R.; Klafter, J. The random walk's guide to anomalous diffusion: a fractional dynamics approach. *Phys. Rep.* **2000**, *339*, 1–77.

(16) Elowitz, M. B.; Surette, M. G.; Wolf, P.-E.; Stock, J. B.; Leibler, S. Protein mobility in the cytoplasm of Escherichia coli. *J. Bacteriol.*. **1999**, *181*, 197–203.

(17) Guo, C.; Luo, Y.; Zhou, R.; Wei, G. Probing the self-assembly mechanism of diphenylalanine-based peptide nanovesicles and nanotubes. *ACS Nano* **2012**, *6*, 3907–3918.

(18) Hoeffding, W. Probability inequalities for sums of bounded random variables. *J. Am. Stat. Assoc.* **1963**, *58*, 13–30.

(19) Swain, M. J.; Ballard, D. H. Color Indexing. *Int. J. Comput. Vision* **1991**, *7*, 11–32.

(20) Prinz, J.-H.; Wu, H.; Sarich, M.; Keller, B.; Senne, M.; Held, M.; Chodera, J. D.; Schütte, C.; Noé, F. Markov models of molecular kinetics: Generation and validation. *J. Chem. Phys.* **2011**, *134*, 174105.
